# Gut microbiota-related metabolite alpha-linolenic acid mitigates intestinal inflammation induced by oral infection with *Toxoplasma gondii*

**DOI:** 10.1186/s40168-023-01681-0

**Published:** 2023-12-12

**Authors:** Jing Yang, Songhao Liu, Qian Zhao, Xiaobing Li, Kangfeng Jiang

**Affiliations:** College of Veterinary Medicine, Yunnan Agricultural University, Kunming, 650201 Yunnan China

**Keywords:** *Toxoplasma gondii*, Intestinal inflammation, Gut microbiota, Metabolite, Alpha-linolenic acid, Fecal microbiota transplantation

## Abstract

**Background:**

Oral infection with cysts is the main transmission route of *Toxoplasma gondii* (*T. gondii*), which leads to lethal intestinal inflammation. It has been widely recognized that *T. gondii* infection alters the composition and metabolism of the gut microbiota, thereby affecting the progression of toxoplasmosis. However, the potential mechanisms remain unclear. In our previous study, there was a decrease in the severity of toxoplasmosis after *T. gondii* α-amylase (α-AMY) was knocked out. Here, we established mouse models of ME49 and Δ*α-amy* cyst infection and then took advantage of 16S rRNA gene sequencing and metabolomics analysis to identify specific gut microbiota-related metabolites that mitigate *T. gondii*-induced intestinal inflammation and analyzed the underlying mechanism.

**Results:**

There were significant differences in the intestinal inflammation between ME49 cyst- and Δ*α-amy* cyst-infected mice, and transferring feces from mice infected with Δ*α-amy* cysts into antibiotic-treated mice mitigated colitis caused by *T. gondii* infection. 16S rRNA gene sequencing showed that the relative abundances of gut bacteria, such as *Lactobacillus* and *Bacteroides*, *Bifidobacterium*, *[Prevotella]*, *Paraprevotella* and *Macellibacteroides*, were enriched in mice challenged with Δ*α-amy* cysts. Spearman correlation analysis between gut microbiota and metabolites indicated that some fatty acids, including azelaic acid, suberic acid, alpha-linolenic acid (ALA), and citramalic acid, were highly positively correlated with the identified bacterial genera. Both oral administration of ALA and fecal microbiota transplantation (FMT) decreased the expression of pro-inflammatory cytokines and restrained the MyD88/NF-κB pathway, which mitigated colitis and ultimately improved host survival. Furthermore, transferring feces from mice treated with ALA reshaped the colonization of beneficial bacteria, such as *Enterobacteriaceae*, *Proteobacteria*, *Shigella*, *Lactobacillus*, and *Enterococcus*.

**Conclusions:**

The present findings demonstrate that the host gut microbiota is closely associated with the severity of *T. gondii* infection. We provide the first evidence that ALA can alleviate *T. gondii*-induced colitis by improving the dysregulation of the host gut microbiota and suppressing the production of pro-inflammatory cytokines via the MyD88/NF-κB pathway. Our study provides new insight into the medical application of ALA for the treatment of lethal intestinal inflammation caused by *Toxoplasma* infection.

Video Abstract

**Supplementary Information:**

The online version contains supplementary material available at 10.1186/s40168-023-01681-0.

## Background

*Toxoplasma gondii* (*T. gondii*) is one of the most widespread obligate intracellular parasites, has a broad host range, and can infect all warm-blooded animals including humans [1]. Toxoplasmosis in immunocompromised and congenitally immunodeficient hosts often leads to severe consequences, including enteritis, encephalitis, neonatal abortion, and even death [2, 3]. The primary route of host infection with *T. gondii* is usually through oral ingestion of contaminated water and undercooked food, followed by invasion and migration to the intestinal epithelial barrier, which is the crucial step for establishing infection and proliferation of the parasite [4]. Bradyzoites are released from the cyst and then rapidly invade the intestinal mucosa to convert to tachyzoites, which eventually reach the nervous system and muscle tissue [5, 6]. In the early period of infection, monocytes and neutrophils are the main cells recruited in the intestine to produce IL-12, activating the secretion of IFN-γ, which restrains parasite growth [7, 8]. However, intestinal tissue may also undergo severe pathological changes due to overproduction of pro-inflammatory cytokines and nitric oxide [4, 9]. It has been reported that oral infection causes loss of Paneth cells, disruption of the epithelial barrier, and leakage of luminal contents into the submucosa to drive intestinal inflammation [10]. Additionally, oral infection lowers the tolerance to antigens in the intestinal lumen, and the presence of commensal microorganisms ultimately triggers a more intense inflammatory response [11, 12].

*T. gondii* continuously secretes relevant factors while invading intestinal tissue, resulting in dramatic changes in the number and composition of gut microbiota [13]. Analysis of the intestinal microbiome of mice orally infected with cysts reveals that ileitis is accompanied by increased bacterial load, decreased species diversity, and bacterial translocation, which is especially characterized by a significant reduction or disappearance of *Bacteroidetes* and *Firmicutes* [14]. In addition, the number of *Escherichia coli* markedly increases due to the loss of Paneth cells and the absence of secreted antimicrobial substances, which leads to bacteria accumulating in the inflamed ileum, translocating to the lamina propria, and then spreading to other tissues [15, 16]. The survival rate of germ-free animals infected with *T. gondii* is significantly increased, suggesting that the gut microbiota can affect the process of toxoplasmosis [14]. The gut microbiota coevolves with the intestinal immune system of the host, which plays an important role in the expression of regulatory immune mediators and in the development, recruitment, and differentiation of immune cells [17]. The metabolites produced by gut microbiota may affect the survival and physiological status of parasites, leading to a different infection course [18]. Previous studies have indicated that joint use of antibiotics can completely eliminate the bacteria in the intestine, so that the generated germ-free mice show no intestinal inflammation reaction after *T. gondii* stimulation [19]. Furthermore, the combined utilization of *Escherichia coli* and *Prevotella* is effective in restoring susceptibility to toxoplasmosis, and mice recolonized with *Lactobacillus johnsonii* also exhibited elevated levels of NO and IFN-γ in the intestine [10, 19]. An increasing body of evidence suggests that the regulation of gut microbiota can be applied to cure parasitosis. Probiotic treatment or fecal microbiota transplantation (FMT) in the host is certified to be beneficial during *T. gondii* infection [20]. However, the current research on the role of gut microbiota and metabolites in the control of *Toxoplasma*-induced intestinal inflammation is still limited.

*T. gondii* α-amylase (α-AMY), the key enzyme in amylopectin metabolism, was proven to be essential for maintaining chronic infection [21, 22]. The function of cysts significantly changed after α-AMY was knocked out, resulting in a decrease in the severity of toxoplasmosis [23], which is increasingly attributed to the gut microbiota [24]. On the basis of existing theories and previous results, mouse models infected with cysts of different virulence were established to explore how gut microbiota affects *T. gondii-*induced intestinal inflammation. In this study, we revealed that Δ*α-amy* cyst infection significantly alleviated intestinal inflammation, which was shown to be closely related to gut microbiota. We then utilized 16S rRNA gene sequencing and metabolomics analysis to identify variations in the gut microbiota composition and metabolism in mice with different intestinal inflammation induced by *T. gondii*. Using Spearman correlation analysis, we identified the gut metabolite alpha-linolenic acid (ALA), which was highly positively correlated with the variational bacterial genera. Finally, orally administered ALA mouse models of *T. gondii*-induced colitis were established to demonstrate that ALA was effective in alleviating intestinal inflammation and improving the dysregulation of gut microbiota. This study provides new insight into the interaction mechanisms between *T. gondii* cysts and gut microbiota and suggests that ALA is a potential drug candidate for the treatment of *T. gondii*-induced intestinal inflammation.

## Methods

### Animals and parasites

Seven-week-old female ICR mice were obtained from the Experimental Animal Center of Kunming Medical University (Kunming, China). Tachyzoites of the wild type (WT) II strain ME49 and Δ*α-amy* mutant were propagated in human foreskin fibroblast cells (obtained from ATCC, Manassas, VA, USA) and then used to infect mice to acquire corresponding cysts.

### Mouse infection and sample collection

Female ICR mice (7 weeks old, *n* = 15) were orally infected with 50 cysts of the ME49 or Δ*α-amy* strain. Subsequently, infection was confirmed by MIC3-based ELISA on serum taken 10 days post-infection, and mice that tested negative were not included in subsequent analyses [22]. Six mice were randomly selected from 11 anti-*Toxoplasma* antibody-positive mice for body weight analysis, and the feces were harvested in sterile tubes on day 10 post-infection and immediately stored at − 80 ℃ for FMT, 16S rRNA gene sequencing, and metabolomics analysis. Next, these mice continued to be monitored daily, for 30 days, to assess the survival rate. For the measurement of cytokine levels and histopathological analysis, the remaining mice (*n* = 5) were sacrificed for the collection of colon segments on day 10 post-infection.

To investigate the relationship between gut microbiota and the severity of *T. gondii* infection. Specific pathogen-free (SPF) mice were randomly divided into two groups and pretreated with antibiotic solution (ATB) before FMT, and the ATB method was performed as previously described [25, 26]. Briefly, mice were given sterile water containing ampicillin (1 mg/mL), streptomycin (5 mg/mL), vancomycin (0.25 mg/mL), and colistin (1 mg/mL) for 3 days. The ATB treatment was discontinued 1 day before FMT. Feces were processed as previously described [26, 27]. Briefly, the fresh fecal samples were homogenized in a sterile environment under N_2_ gas within 2 h of collection, passed through 0.25-mm stainless cell strainers, and then centrifuged at 6000 × *g* for 15 min to remove impurity particles. One day after FMT, mice were orally infected with 50 ME49 cysts. The treatment groups were as follows: (1) ME49-FMT group: FMT of feces from ME49-infected mice and followed by infection with ME49 cysts; and (2) Δ*α-amy*-FMT group: FMT of feces from Δ*α-amy-*infected mice followed by infection with ME49 cysts. Six mice were randomly selected for body weight and survival analysis, and colon tissues from other mice (*n* = 5) were collected on day 10 post-infection for subsequent analysis.

To study the effect of ALA on *T. gondii*-induced colitis, mice pretreated with ATB were randomly divided into two groups and were orally administered 1 g/kg of body weight ALA (Sigma-Aldrich, St. Louis, MO, USA) or equal phosphate-buffered saline (PBS) for 3 days [28]. Subsequently, mice were challenged with ME49 cysts on day 0 to induce colitis, and mice that tested negative were not included in subsequent analyses. The treatment groups were as follows: (1) PBS group: oral gavage of PBS for 3 days and then infection with ME49 cysts, and (2) ALA group: oral gavage of ALA for 3 days and then infection with ME49 cysts. Mice (*n* = 6) were used to record body weight and survival rate. The remaining mice (*n* = 5) were euthanized on day 10, and the colon tissues were harvested for subsequent analysis. For fecal transplantation from the PBS and ALA groups, ATB-pretreated mice were randomly divided into two groups and received fecal transplantation one day before infection with 50 ME49 cysts. The treatment groups were as follows: (1) PBS-FMT group: FMT of feces from the PBS group followed by infection with ME49 cysts, and (2) ALA-FMT group: FMT of feces from the ALA group followed by infection with ME49 cysts. Six mice were used to analyze body weight, survival rate, and 16S rRNA gene sequencing, and the colon tissues from the remaining 5 mice were randomly collected on day 10 post-infection for the detection of inflammatory indicators. All experiments were performed independently three times.

### DNA extraction and 16S rRNA gene sequencing

Bacterial genomic DNA from fecal samples was extracted using QIAamp DNA Isolation Kits (Qiagen, Hilden, Germany). Subsequently, DNA concentration was quantified by Nanodrop (Thermo Fisher Scientific, Fair Lawn, NJ, USA), and DNA quality was measured using 1.2% agarose gel electrophoresis. The V3–V4 hypervariable region of the 16S rRNA gene was amplified via PCR using specific barcode primers (338F: 5′- ACTCCTACGGGAGGCAGCAG-3′, 806R: 5′-GGACTACHVGGGTWTCTAAT-3′) to characterize the taxonomic profile of the mouse gut microbiome. The amplicon DNA was purified via Vazyme VAHTSTM DNA Clean Beads (Vazyme, Nanjing, China), and the PCR products were quantified using a Quant-iT PicoGreen dsDNA Assay Kit (Microplate reader, BioTek, FLx800). The TruSeq Nano DNA LT Library Prep Kit (Illumina) was applied to prepare a PCR product library, which was then sequenced by double-end sequencing on the Illumina MiSeq platform (600 cycles).

### Sequencing data analysis

The original high-throughput sequencing data were preliminarily screened according to sequence quality, and the problem samples were retested. The library and samples were divided based on the index and barcode information of the original sequence by quality primary screening, and then the barcode sequence was removed. Next, sequencing data were processed using QIIME2, version 2020.02 [29]. The DADA2 plugin (version 3.11) was used for chimera and singleton removal, quality filtering, denoising, and splicing to obtain the amplicon sequence variant (ASV) feature table [30]. Sequences with more than two expected errors were removed by the DADA2 fastqPairedFilter and fastqFilter functions, and the first 20 nucleotides and the last 10 nucleotides were trimmed according to the quality characteristics of the data. The search command fastq_mergepairs with a minimum overlap of 20 bases and a maximum difference of 1 base was applied to merge the forward and reverse reads for denoising using the parameters: -p-trunc-len 150. The cluster_size module was used to cluster high-quality sequences at a 97% similarity level to obtain ASVs with the following parameters: -id 0.9 -sizein -sizeout -fasta_width 0. The classify-sklearn algorithm of QIIME2 was used for each ASV feature sequence [31] and the species were annotated via a pretrained naive Bayes classifier and the default parameters: -gg-13–8-99–515-806-nb-classifier.qza. The alpha diversity level of each sample was evaluated on the basis of the distribution of ASV/OUT, which was performed with the following parameters: -p-steps 10 -p-min-depth 10 -p-iterations 10, -p-max-depth 95%. Beta diversity analysis was performed by Jaccard, Bray–Curtis, unweighted UniFrac and weighted UniFrac outputs to assess the difference between groups using PERMANOVA in R software (version 2.15.3, vegan package). In addition, the linear discriminant analysis effect size (LEfSe, http://huttenhower.sph.harvard. edu/galaxy/root?tool_id = lefse_upload) with various parameters was used to detect differentially abundant taxa across groups using the default parameters for linear discriminant analysis (LDA > 2 and *p* < 0.05). Kyoto Encyclopedia of Genes and Genomes (KEGG) functional annotation (www.kegg.jp/kegg) was applied to predict the metabolic functions and differential pathways of gut microbiota using default parameters, with the exception of -k 50 -sensitive -e 0.00001 [32]. Spearman correlation was used to analyze the correlations between the microbial taxa and phenotypic variables by R software.

### Untargeted metabolomics analysis

The untargeted metabolomics analysis was conducted as previously described [33]. Briefly, 5 mg of lyophilized feces was thawed in an ice bath to diminish degradation, and 120 μL of methanol containing internal standard was added to extract the metabolites. After homogenization, the sample was centrifuged at 18,000 × *g* for 20 min, and then 20 μL of freshly prepared derivative reagents was added to a 96-well plate, which was sealed at 30 ℃ for 60 min. After derivatization, 330 μL of ice-cold 50% methanol solution was used to dilute the sample. Subsequently, the sample in each well was stored at − 20 ℃ for 20 min, followed by 4000 × *g* centrifugation at 4 ℃ for 30 min. The supernatant was collected and transferred to a new 96-well plate with 10 μL internal standards, and serial dilutions of derivatized stock standards were added to the left wells. Ultimately, the samples were sealed for the following LC–MS analysis.

An ultra-performance liquid chromatography coupled to tandem mass spectrometry (UPLC-MS/MS) system (ACQUITY UPLC-Xevo TQ-S, Waters Crop., Milford, MA, USA) was used to quantitate the metabolites. The treated analytes were separated in mobile phases containing water with 0.1% formic acid and acetonitrile/IPA (70:30) by an ACQUITY UPLC BEH C18 1.7 μM VanGuard precolumn (2.1 × 5 mm) and analytical column (2.1 × 100 mm), and the flow rate of the mobile phases was set to 0.40 mL/min. Moreover, the source and desolvation temperatures were respectively set at 150 ℃ and 550 ℃, respectively, and 1000 L/Hr desolvation gas flow was performed to acquire metabolite profiles.

The raw data files produced by UPLC-MS/MS were processed using TMBQ software (v1.0, Metabo-Profile, Shanghai, China) to perform peak integration, calibration, and quantitation for each metabolite. The self-developed platform iMAP (v1.0, Metabo-Profile, Shanghai, China) was utilized for statistical analyses, including principal component analysis (PCA), partial least square-discriminant analysis (PLS-DA), univariate analysis, and pathway analysis. For metabolomics analysis, multivariate statistical analyses such as PCA and PLS-DA, and univariate statistical analyses (Student’s *t* test, Mann–Whitney-Wilcoxon *U* test, analysis of variance [ANOVA], and correlation analysis) were performed. The uniform calculation method was adapted from the statistical analysis package widely used in R studio (http://cran.r-project.org/). Spearman correlation analysis was used to analyze the correlations between the microbial taxa and metabolites.

### Histopathological analysis

Colon tissues were collected from each group on day 10 post-infection and fixed with 4% paraformaldehyde overnight. Then, the colon tissues were made into 4-µm-thick sections, and stained with hematoxylin and eosin (H&E). The histopathological changes were evaluated with light microscopy (Olympus, Japan). The sections were scored by two blinded investigators for histological evidence of intestinal damage with a scoring system described previously [34]. Briefly, the amount and depth of inflammation in the sections was evaluated using a 0 to 3 score range and the extent of crypt damage was assessed using a 0 to 4 score range. Each section was then scored separately by creating the product of the grade for that feature and the percentage involvement.

### ELISA

Tissues were washed with a 10-weight volume of precooled PBS, and then PBS was added to the homogenized tissues. The prepared homogenate was centrifuged at 12,000 × *g* for 10 min at 4 ℃, and the supernatant was used for cytokine detection. The pro-inflammation cytokine production was measured using mouse IFN-γ (Servicebio, Wuhan, China), TNF-α, IL-1β, and IL-6 ELISA kits (Meimian Technology, Jiangsu, China) according to the manufacturer’s protocols. Briefly, 100 μL of standard substance and tissue samples was added to appropriate wells at 37 ℃ for 1 h, respectively. Subsequently, 100 μL of detection antibodies was added to each well and incubated at 37 ℃ for 1 h. After washing 5 times, 100 μL of avidin-HRP solution was incubated at 37 ℃ for 30 min. Finally, 100 μL of TMB substrate and stop solution were incubated to visualize the immunoreaction, and the *OD* value was determined at 450 nm using a microplate reader.

### Immunofluorescence

After dewaxing paraffin sections to water, the sections were repaired with EDTA buffer (pH = 8.0) and washed with PBS three times. The paraffin sections were blocked in 10% BSA at 37 ℃ for 30 min, the blocking buffer was removed and the paraffin sections were incubated at 4 ℃ overnight with primary antibodies against MyD88 (1:100, Servicebio, Wuhan, China). Subsequently, the sections were washed with PBS three times and incubated with secondary antibodies (1:100, Servicebio, Wuhan, China) at room temperature for 50 min. DAPI was added and incubated at room temperature in the dark for 10 min to counterstain the cell nuclei. Finally, the paraffin sections were sealed with fluorescent mounting media for further analysis. After staining, fluorescence microscope coupled with the MicroPublisher imaging software platform (Q-imaging) was used to observe the fluorescence intensity.

### Western blot analysis

Total protein was extracted from colon tissues using RIPA reagent (Biosharp, Beijing, China) according to the manufacturer’s protocols, and then the protein concentrations were measured using the BCA protein assay kit (Beyotime, Shanghai, China). Equal amounts of proteins (50 μg) were separated by 10% SDS-PAGE prior to transferring to PVDF membranes (Millipore, Massachusetts, USA), and then the PVDF membranes were blocked in 5% BSA at room temperature for 2 h. The membranes were incubated with primary antibodies against NF-κB p65, occludin (1:2000 dilutions, Abcam, Cambridge, UK), phospho-NF-κB p65, and β-actin (1:1000 dilutions, CST, MA, USA) at 4 ℃ overnight. Subsequently, the membranes were washed with TBST five times and detected with secondary antibodies (1:5000 dilutions; Thermo Fisher Scientific, Fair Lawn, NJ, USA) at room temperature for 1 h. Eventually, the protein levels were scanned by an ECL Plus Western blotting Detection System (GE Healthcare, USA). The ray values of protein bands were measured by Image Pro Plus 6.0 software (Media Cybernetics, Silver Spring, MD, USA), and the relative expression levels of corresponding proteins were normalized to β-actin, which was used as a control.

### Statistical analysis

Analyses and graphics were acquired with GraphPad Prism 6 (GraphPad Software Inc., La Jolla, CA, USA), and all data were presented as the mean ± SEM. The significant differences between groups were determined using Student’s* t* test as indicated in the figure legends, cumulative mortality was graphed as Kaplan–Meier survival plots and analyzed by the Mantel-Cox log-rank test, and body weight changes were determined using two-way ANOVA. A value of *p* < 0.05 was defined as statistically significant.

## Results

### Clinical signs and colon histopathological changes are different in mice infected with ME49 or Δ*α-amy* cysts

**Fig. 1:**
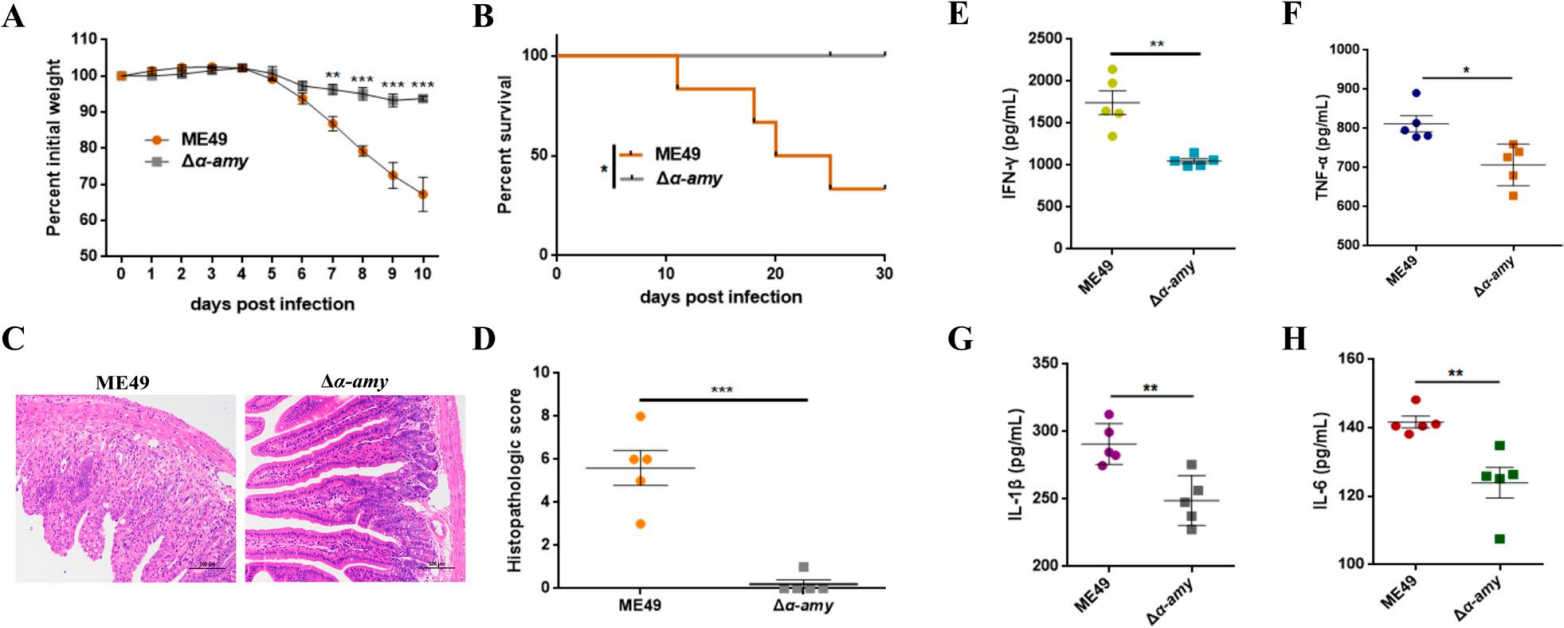
Oral infection with Δ*α-amy* cysts significantly alleviated *T. gondii*-induced colitis. **A** Body weight changes of mice infected with cysts of the indicated strains for 10 days. Statistical significance was determined using two-way ANOVA. ***p < 0.001, **p < 0.01 compared with the ME49 group. **B** The survival curves of mice infected with cysts of indicated strains. ME49 and Δ*α-amy* cysts were respectively used to infect ICR mice (50 cysts per mouse, 6 mice for each group) by oral injection, and the survival of mice was monitored for 30 days. The survival rate was determined using the log-rank (Mantel-Cox) test. *p < 0.05 compared with the ME49 group. **C**, **D** H&E staining of the mice (*n* = 5 per group) colon tissue samples (Scale bars = 100 μm, × 20 objective) and histological scores of colonic tissues. Representative images are shown. **E**, **H** The levels of IFN-γ, TNF-α, IL-1β, and IL-6 were measured by ELISA (*n* = 5 per group). The data are presented as the means ± SEM. Statistical significance was determined with Student’s* t* test. ***p < 0.001, **p < 0.01, *p < 0.05. All experimental data are representative of three independent experiments with similar results

To explore whether different cyst infections may trigger disparate effects on intestinal inflammation, cysts of the ME49 and Δ*α-amy* strains were harvested and intragastrically administered to 7-week-old mice. The percentile curves for the mouse body weights after 10 days of infection revealed that mice infected with ME49 cysts lost more weight than those infected with Δ*α-amy* cysts, demonstrating that cysts of the Δ*α-amy* strain were less damaging to the host (Fig. 1A). After 30 days of continuous infection, the survival rate of mice infected with ME49 cysts was 33.33%. Nevertheless, 100% survival rate and significantly reduced cysts were observed in mice infected with Δ*α-amy* cysts (Fig. 1B, Fig. S1), which showed no obvious clinical symptoms in the form of locomotor activity and depressive symptoms. The above results indicated that Δ*α-amy* cysts were avirulent to mice, consistent with the results we reported previously [23]. Murine colon tissues were collected to measure histopathological changes, and the results from Fig. 1C, D manifested that the mice infected with ME49 cysts showed structural destruction of intestinal villi, massive infiltration of inflammatory cells, and swelling of the mesentery, while the mice infected with Δ*α-amy* cysts exhibited normal colon structure and few obvious inflammatory lesions. Moreover, the levels of pro-inflammatory cytokines IFN-γ, TNF-α, IL-1β, and IL-6 were significantly lower in the Δ*α-amy* group, further suggesting that infection with Δ*α-amy* cysts hardly caused remarkable inflammatory damage to intestinal tissues (Fig. 1E–H). These results demonstrated that there were significant differences in the severity of intestinal inflammation between mice infected with ME49 and Δ*α-amy* cysts.

### The transfer of fecal microbiota from mice infected with Δ*α-amy* cysts increases the resistance to colitis induced by *T. gondii* infection

**Fig. 2:**
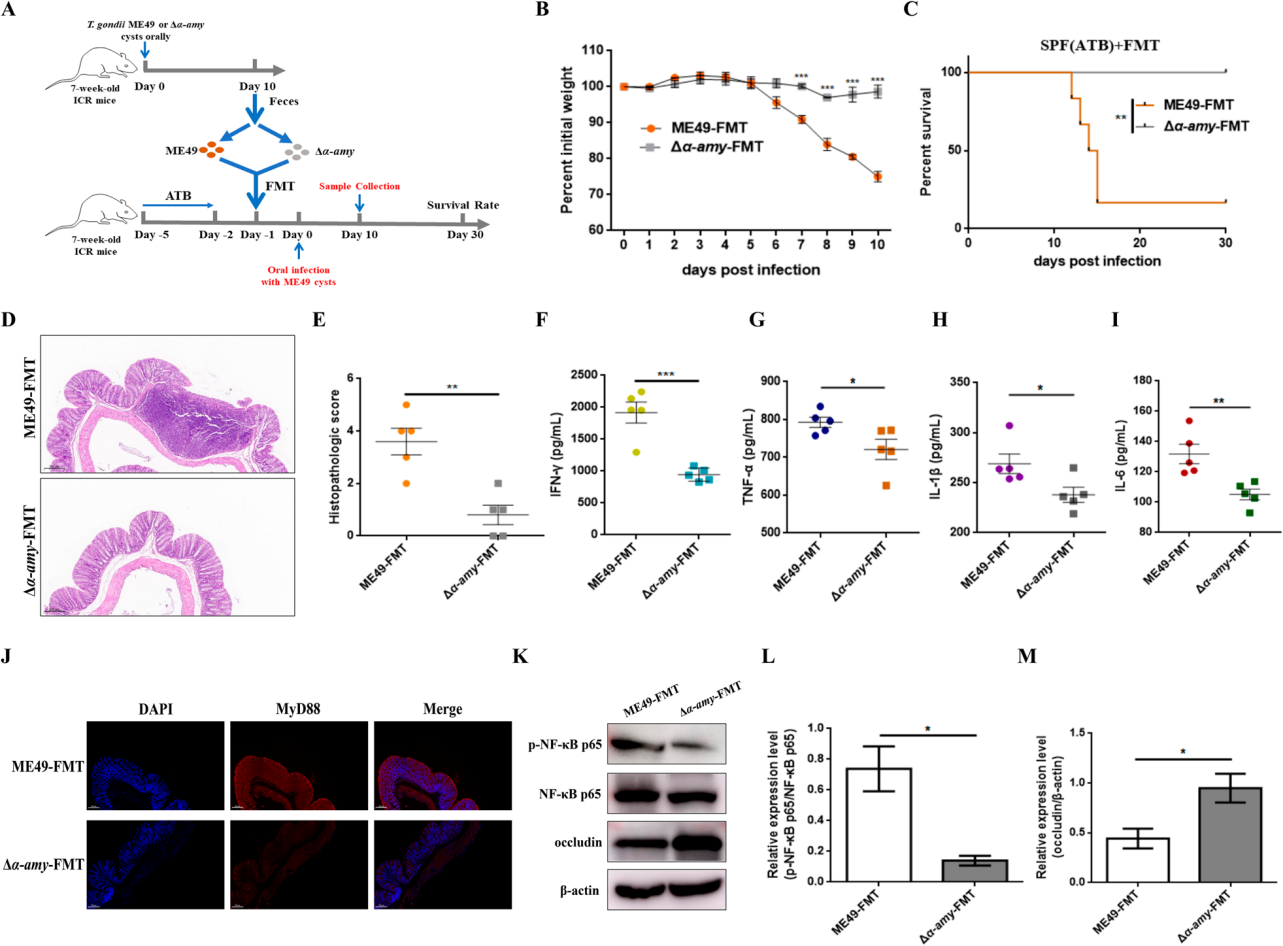
The severity of cyst infection is closely related to the gut microbiota. **A** Illustration of FMT experiments. Feces were collected from mice infected with ME49 or Δ*α-amy* cysts on days 10. SPF mice that had been treated with an antibiotic solution (ATB) for 3 days underwent FMT. One day after FMT, the mice were infected with 50 cysts of ME49. **B** Body weight changes of mice infected with cysts of the indicated strains for 10 days. Statistical significance was determined using two-way ANOVA. ***p < 0.001 compared with the ME49-FMT group. **C** Survival curves of the ME49-FMT and Δ*α-amy*-FMT groups. ME49 cysts were used to infect ICR mice that underwent FMT from mice infected with ME49 or Δ*α-amy* (50 cysts per mouse, 6 mice for each group), respectively. The survival of mice was monitored for 30 days, and the survival rate was determined using the log-rank (Mantel-Cox) test. **p < 0.01 compared with ME49-FMT group. **D**, **E** H&E staining of the mouse (*n* = 5 per group) colon tissue samples (Scale bars = 200 μm, × 10 objective) and histological scores of colonic tissues. Representative images are shown. **F**–**I** The levels of IFN-γ, TNF-α, IL-1β, and IL-6 were measured by ELISA (*n* = 5 per group). Statistical significance was determined with Student’s* t* test. **p < 0.01, *p < 0.05. **J** The expression level of MyD88 was measured by immunofluorescence. MyD88 protein was labelled with a red fluorophore, and the cell nucleus was labelled with a blue fluorophore (Scale bars = 200 μm, × 10 objective). Representative images are shown. **K**–**M** The expression levels of p-NF-κB p65 and occludin were measured by Western blotting. β-actin was used as a control. The data are presented as the means ± SEM. Statistical significance was determined with Student’s* t* test. *p < 0.05. All experimental data are representative of three independent experiments with similar results

To investigate whether the differences in the inflammatory responses of mice infected with different cysts are related to gut microbiota, we performed FMT in antibiotic-treated mice (Fig. 2A). The weight loss analysis was performed to evaluate the clinical symptoms of each group, and the results obtained from Fig. 2B indicated that mice that received the fecal microbiota of mice infected with Δ*α-amy* cysts tended to alleviate weight loss. Nevertheless, the body weight of mice in the ME49-FMT group was significantly lower than that of mice in the Δ*α-amy*-FMT group from day 7. The survival rate of the remaining mice in the ME49-FMT and Δ*α-amy*-FMT groups was determined after 30 days of continuous infection. The ME49-FMT group on day 10 post-infection led to an 83.3% mortality rate after *T. gondii* infection, while the mortality rate of the Δ*α-amy*-FMT group was 0% (Fig. 2C). Furthermore, mucosal damage and inflammatory cell infiltration in the Δ*α-amy*-FMT group were significantly alleviated compared with those in the ME49-FMT group (Fig. 2D, E). Measurement of inflammatory cytokines showed that FMT of the fecal microbiota from Δ*α-amy* mice significantly decreased the levels of IFN-γ, TNF-α, IL-1β, and IL-6 (Fig. 2F–I). Previous studies have highlighted the importance of MyD88 signalling in Th1-type cytokine production. Although it is essential for host survival, MyD88 signalling activation can lead to the overproduction of pro-inflammatory cytokines, which in turn can cause severe inflammatory responses [35]. Therefore, immunofluorescence was applied to detect the expression of MyD88 in colon tissues. As shown in Fig. 2J, the expression of MyD88 was notably decreased in the Δ*α-amy*-FMT group compared with that in the ME49-FMT group. NF-κB is a crucial downstream molecule of MyD88, and nuclear translocation of NF-κB p65 induces the transcription of inflammatory cytokines, including TNF-α, IL-1β, and IL-6 [36]. The results showed that the transplantation of fecal microbiota from Δ*α-amy* mice markedly reduced the expression level of p-NF-κB p65 (Fig. 2K, L). Moreover, the level of the cell tight-junction-related protein occludin, in mice treated with fecal microbiota harvested from Δ*α-amy* mice, was observably upregulated (Fig. 2K, M). Overall, these data illuminated that the fecal microbiota of mice infected with Δ*α-amy* cysts was likely to contain specific intestinal bacteria that can resist *T. gondii*-induced intestinal inflammation.

### Gut microbiota between mice infected with ME49 and Δ*α-amy* cysts exhibit distinct differences

**Fig. 3:**
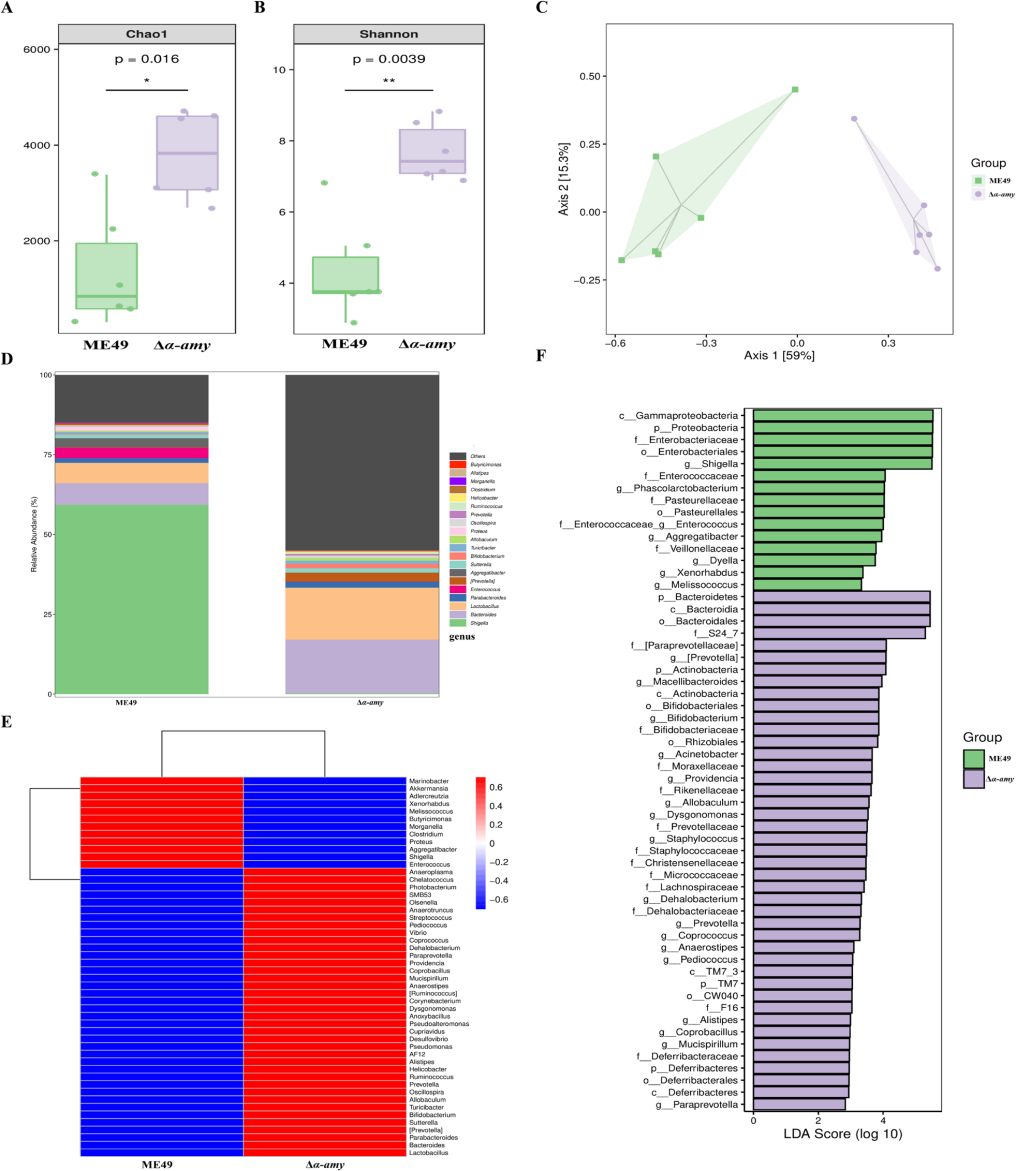
Effect of Δ*α-amy* cyst infection on gut microbiota assembly. The feces samples (*n* = 6 per group) for 16S rRNA sequencing analysis were collected on day 10 post-infection in the experiment shown in Fig. 2. **A** Microbial community abundance (measured by Chao1 index). **B** Microbial community diversity (measured by Shannon index). **C** Principal coordinate analysis (PCoA) based on the weighted UniFrac distance matrix. **D** Relative abundances of fecal bacteria at the genus level. **E** Heatmap of the top 50 microbiota at the genus level in fecal samples from mice infected with ME49 or Δ*α-amy* cysts. Colour indicates the relative microbiota abundances in the group samples; the corresponding relationship between the colour gradient and the value is shown in the gradient colour block. **F** Differentially abundant taxa of fecal microbiota between ME49 and Δ*α-amy* cyst-infected mice was analyzed by LEfSe. LDA score ≥ 2

To identify the critical gut microbiota participating in the resistance to *T. gondii* cyst infection, we utilized 16S rRNA gene sequencing to analyze the fecal microbiota of mice infected with ME49 and Δ*α-amy* cysts on day 10 post-infection. Alpha diversity was detected with the Chao1 and Shannon indices between the two groups. Analysis of the Chao1 diversity index analysis revealed that the total microbiota abundance in the Δ*α-amy* group was significantly higher than that of mice infected with ME49 cysts (*P* = 0.016), and the Shannon diversity index analysis indicated that the microbiota diversity of the Δ*α-amy* group was also obviously higher than that of the ME49 group (*P* = 0.0039) (Fig. 3A, B). Furthermore, principal coordinate (PCoA) of weighted UniFrac distances, principal component analysis (PCA), and nonmetric multidimensional scaling (NMDS) were performed to exhibit the dispersed distribution of data points on the plots of the ME49 and Δ*α-amy* groups, suggesting that the compositions of the fecal microbiota differ considerably between the two groups (Fig. 3C, Fig. S2). At the phylum level, *Bacteroidetes*, *Proteobacteria*, and *Firmicutes* were the preponderant phyla in both the ME49 and Δ*α-amy* groups, and *Bacteroidetes* was enriched in the Δ*α-amy* group, while *Proteobacteria* was decreased (Fig. S3A). At the family level, the relative abundance of *Enterobacteriaceae*, of which most bacteria were gram-negative, was higher in the ME49 group (Fig. S3B). At the genus level, 50 genera of the ME49 group were distinctly different from those of the Δ*α-amy* group, particularly *Lactobacillus*, *Bacteroides*, and *[Prevotella]*, which were more abundant in the Δ*α-amy* group (Fig. 3D, E). We further performed LEfSe analysis to identify the differentially abundant fecal bacterial taxa, and the results revealed that multiple gram-negative bacterial genera, such as *Bifidobacterium*, *[Prevotella]*, *Paraprevotella*, and *Macellibacteroides*, were enriched in the Δ*α-amy* group compared with those in the ME49 group (Fig. 3F). KEGG enrichment pathway analysis identified that biological metabolic pathways, such as infectious diseases and carbohydrate, amino acid, and lipid metabolism, were enriched in differentially expressed species (Fig. S4). These aforementioned results demonstrated that the microbial community structure was considerably differentiated between the ME49 and Δ*α-amy* groups.

### Fecal metabolite alpha-linolenic acid is closely associated with resistance against *T. gondii* cyst infection-induced intestinal inflammation

**Fig. 4:**
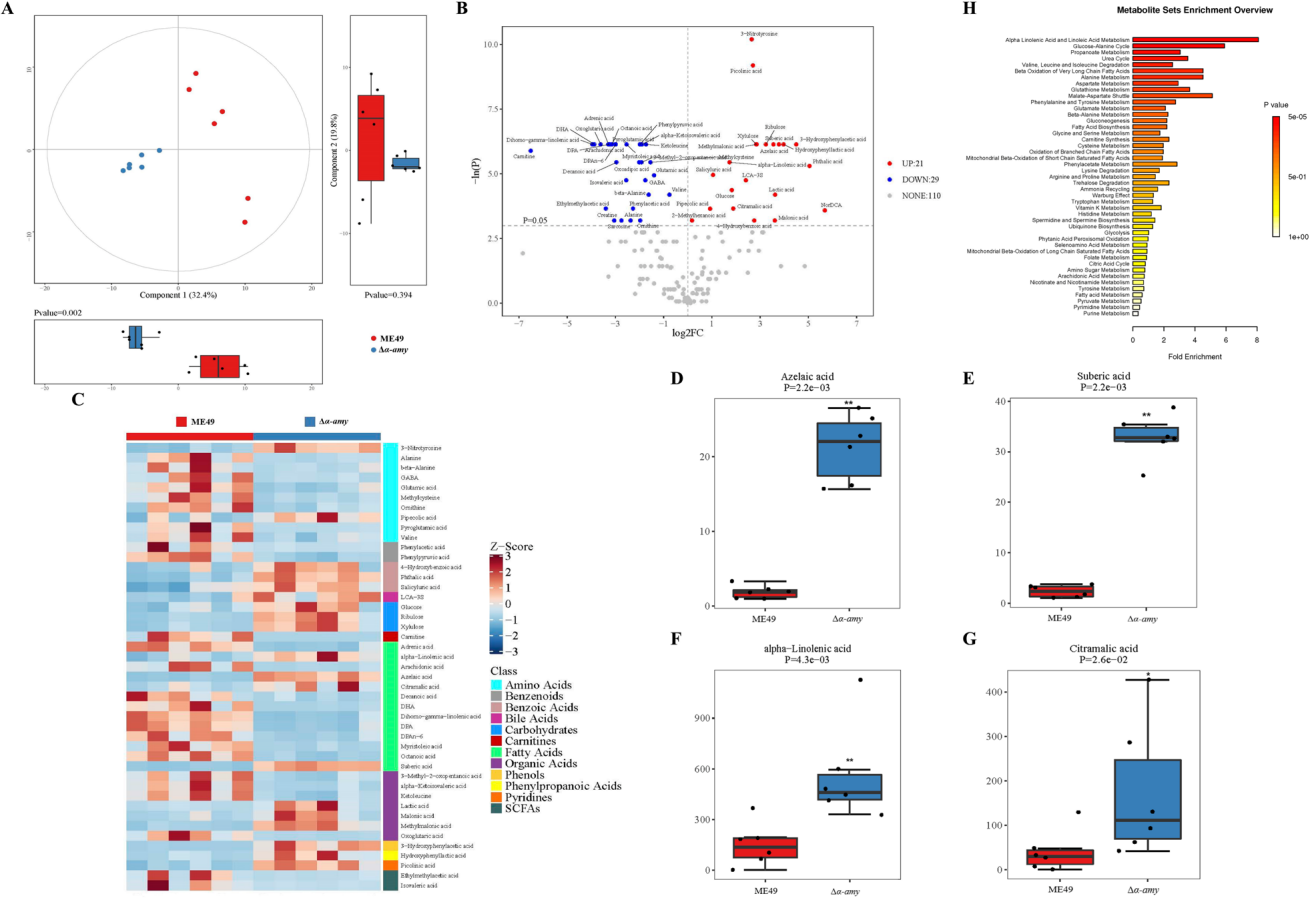
Changes in the fecal metabolomic profiles between ME49 and Δ*α-amy* cysts-infected mice. **A** The fecal metabolomic profiles were clustered using PLS-DA. Data are presented as the mean ± SEM. *P* values were determined using the nonparametric Kruskal–Wallis test. **B** Volcano map analysis of differential metabolites. **C** Relative abundances of metabolites were clustered using a UPGMA dendrogram and shown in a heatmap. The metabolite variation is shown using Z_Score. **D**–**G** The concentrations of fecal azelaic acid, suberic acid, alpha-linolenic acid (ALA), and citramalic acid were displayed as box and dot plots. The data are presented as the mean ± SEM. *P* values were determined using the nonparametric Kruskal–Wallis test. **p < 0.01, *p < 0.05. **H** SMPDB pathway enrichment analysis according to the markedly altered metabolites

**Fig. 5:**
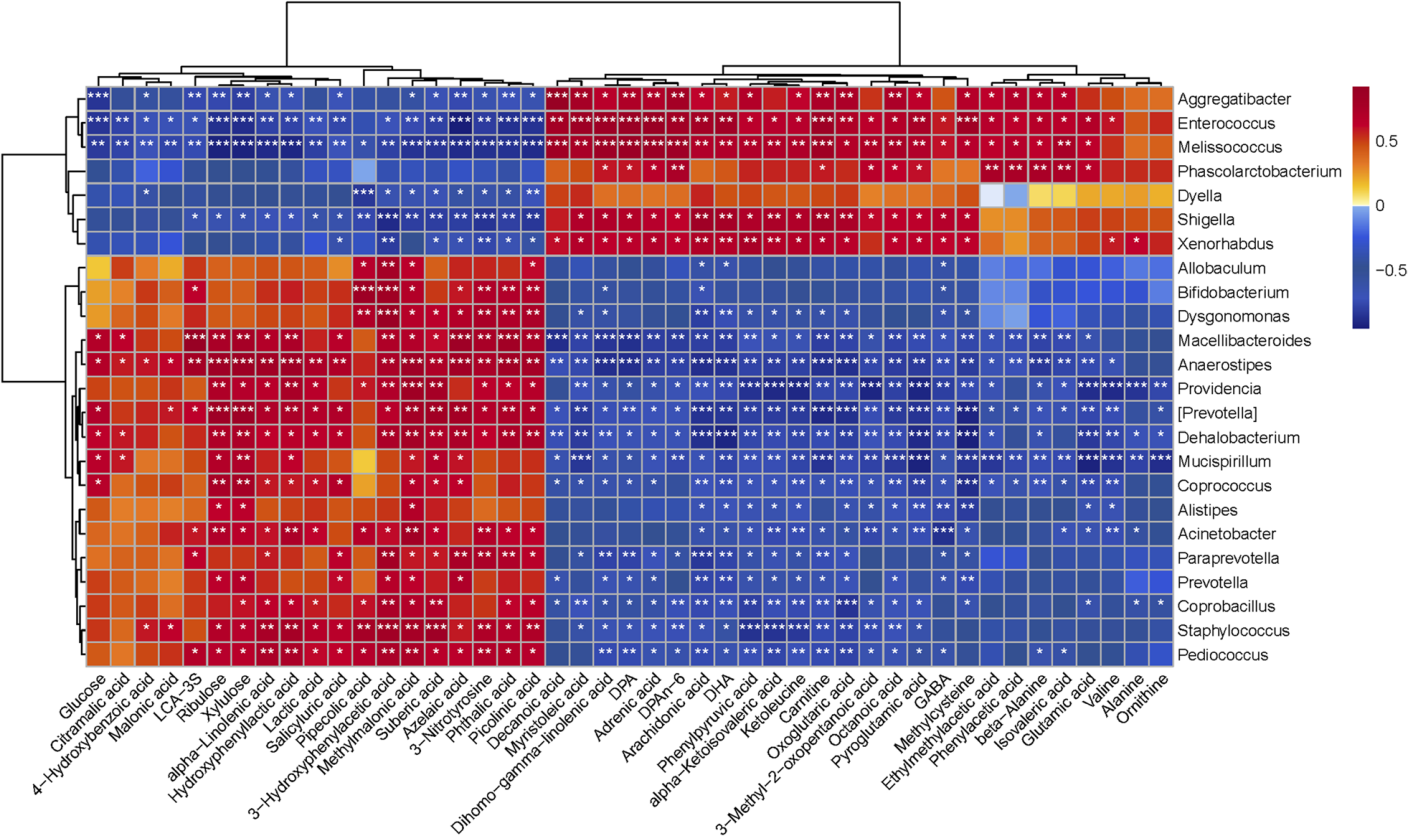
Heatmap of Spearman correlation between gut microbiota and metabolites. The relevance between differentially enriched bacteria and metabolites from mice infected with Δ*α-amy* cysts was interpreted using Spearman correlation analysis. The colors range from blue (negative correlation) to red (positive correlation). ***p < 0.001, **p < 0.01, *p < 0.05

Gut microbiota-associated metabolites can influence intestinal function, especially in improving gut inflammation [37, 38]. Therefore, we used untargeted metabolomics based on the UPLC-MS/MS method to screen and assess the potential metabolites against *T. gondii* infection. Metabolite classification revealed that 26.97% of the compounds were SCFAs, 21.53% were organic acids, 20.59% were amino acids, and 15.78% were fatty acids (Fig. S5A). Analysis of PLS-DA showed that the data point distribution of the ME49 group was distinct from that of the Δ*α-amy* group (Component 1, *p* = 0.002; Component 2, *p* = 0.394; Fig. 4A, Fig. S5B). In addition, PCA based on the Bray–Curtis distance exhibited significant differences in the metabolites identified from the ME49 and Δ*α-amy* groups, consistent with the results of PLS-DA analyses (PC1, *p* = 0.002; PC2, *p* = 0.065; Fig. S5C, D). The volcano diagram showed the expression of differential metabolites between the two groups, and 21 upregulated and 29 downregulated metabolites were identified in the Δ*α-amy* group compared with those in the ME49 group (Fig. 4B). The potential biomarker was screened for metabolites with significant differences in relative quantities, and the results visualized by heatmap indicated that 45 metabolites identified as potential biomarkers were listed and clustered in Fig. 4C. Fatty acids, which have been demonstrated to have anti-inflammatory effects in previous studies, were the most abundant among the identified potential metabolites [39]. The concentrations of several fatty acids (azelaic acid, suberic acid, alpha-linolenic acid, and citramalic acid) were observably higher in the Δ*α-amy* group than in the ME49 group (Fig. 4D–G). The results of the enrichment analysis of pathway-associated metabolite sets (SMPDB) database visualized by bubble chart showed that these differential metabolites were abundant in fatty acid-related metabolisms, such as alpha-linolenic acid and linoleic acid metabolism, beta oxidation of very long-chain saturated fatty acids, oxidation of branched chain fatty acids, and fatty acid biosynthesis (Fig. 4H). Commensal bacteria, including *Lactobacillales* and *Bacteroidetes*, have been proven to produce long-chain fatty acids derived from gut microbiota, which have strong anti-inflammatory effects [40, 41]. Spearman correlation analysis was used to elucidate the correlation between differentially enriched bacteria and heterogeneous metabolites. ALA, a kind of fatty acid significantly enriched in the Δ*α-amy* group, is the gut-derived metabolite that we focused on and is able to reduce intestinal inflammation [28, 42, 43]. ALA was intensively positively correlated with *[Prevotella]* (*R* = 0.73, *P* = 0.01), *Paraprevotella* (*R* = 0.59, *P* = 0.0045), and *Macellibacteroides* (*R* = 0.70, *P* = 0.01). Furthermore, these bacteria were similarly positively correlated with other fatty acids differentially enriched in the Δ*α-amy* group (Fig. 5). All the above results signified that mice infected with Δ*α-amy* cysts may affect the structure of commensal microbiota and promote the uptake of alpha-linolenic acid, thus suppressing the strong inflammatory response in the intestine.

### Oral administration of ALA attenuates colitis induced by *T. gondii* infection

**Fig. 6:**
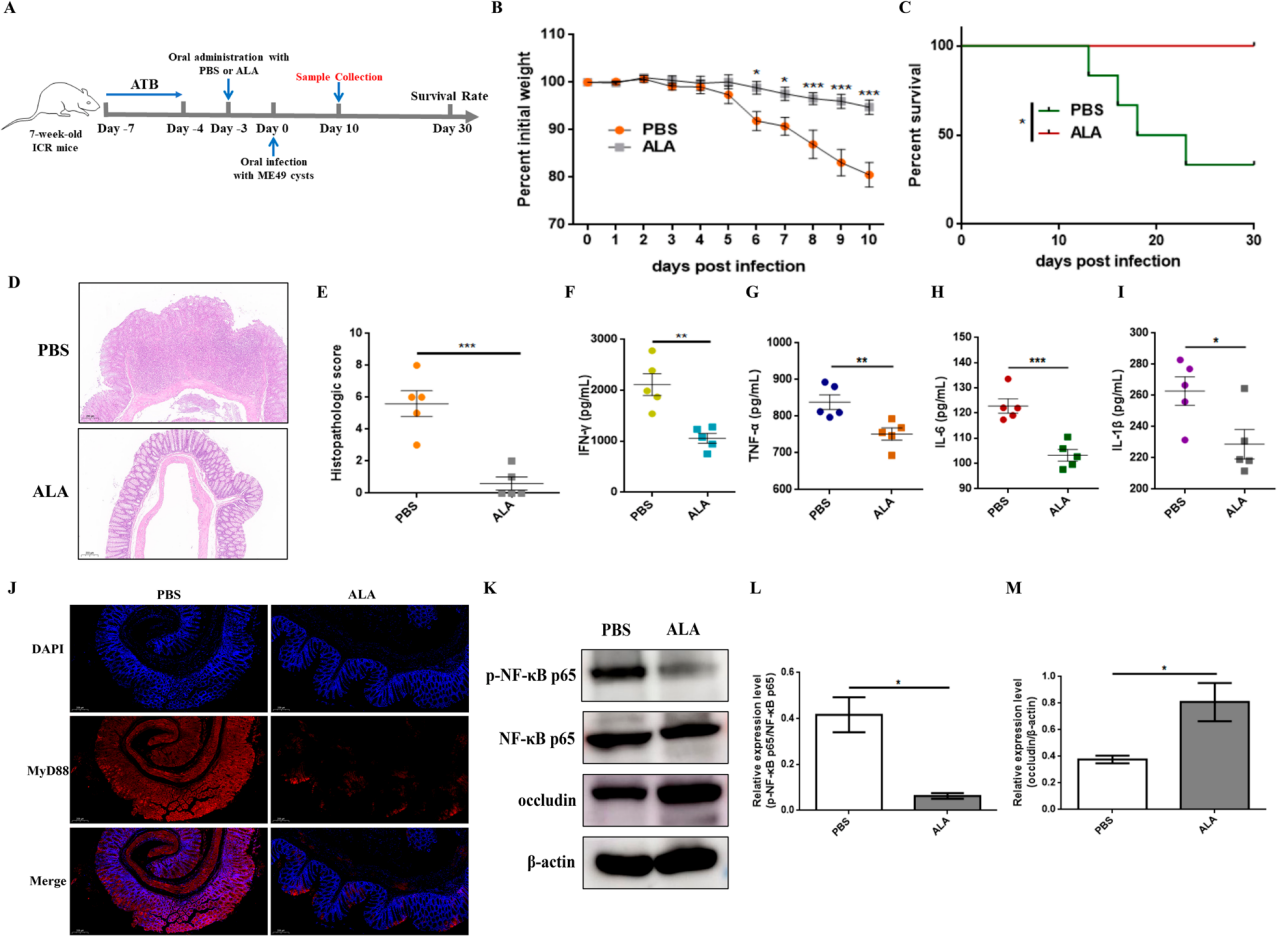
The oral administration of ALA mitigated colitis induced by *T. gondii* cyst infection. **A** Experimental diagram of the mouse infection model. SPF mice were treated with an antibiotic solution (ATB) for 3 days before ALA treatment. One day after ALA treatment, the mice were infected with 50 cysts of ME49. **B** Body weight changes and **C** survival curves of mice (*n* = 6 per group). Statistical significance was determined using two-way ANOVA. ***p < 0.001, *p < 0.05 compared with the PBS group. The survival rate was determined using the log-rank (Mantel-Cox) test. *p < 0.05 compared with the PBS group. **D**, **E** H&E staining of the mouse (*n* = 5 per group) colon tissues (scale bars = 200 μm, × 10 objective) and histological scores of colonic tissues. Representative images are shown. **F**–**I** The levels of IFN-γ, TNF-α, IL-1β, and IL-6 were measured by ELISA (*n* = 5 per group). Statistical significance was determined with Student’s* t* test. ***p < 0.001, **p < 0.01, *p < 0.05. **J** The expression level of MyD88 was measured by immunofluorescence. MyD88 protein was labelled with a red fluorophore, and the cell nucleus was labelled with a blue fluorophore (scale bars = 200 μm, × 10 objective). Representative images are shown. **K**–**M** The expression levels of p-NF-κB p65 and occludin were measured by Western blotting. β-actin was used as a control. The data are presented as the means ± SEM. Statistical significance was determined with Student’s* t* test. *p < 0.05. All experimental data are representative of three independent experiments with similar results

To verify whether ALA can alleviate intestinal inflammation caused by *T. gondii* infection, SPF mice pretreated with antibiotics were orally administered ALA for 3 days and then challenged via oral gavage with PBS or ME49 cysts (Fig. 6A). An obvious decrease in body weight of the PBS group administered on day 6 post-infection, while the body weight of the ALA group showed no significant fluctuation throughout the observation period (Fig. 6B). Furthermore, oral administration of ALA markedly increased the survival rate of recipient mice compared with that of the PBS group (Fig. 6C). Intestinal histopathological analysis revealed that the PBS group showed significant histological changes characterized by structural destruction of intestinal villi and inflammatory cell infiltration, whereas there were only slight lesions in the ALA group (Fig. 6D, E). The production of pro-inflammation cytokines was measured to further assess the inflammatory responses. As shown in Fig. 6F–I, the levels of IFN-γ, TNF-α, IL-1β, and IL-6 were markedly decreased after ALA treatment. To explore whether the reduction in intestinal inflammation by ALA is mediated by MyD88, immunofluorescence was used to measure the expression levels of MyD88 in colon tissues. Compared with that in the PBS group, the expression of MyD88 in the ALA group exhibited a noteworthy decrease (Fig. 6J). Next, the activation of the NF-κB pathway was assessed to investigate the anti-inflammatory mechanism of ALA in *T. gondii-*induced colitis. The results indicated that the expression levels of phosphorylated NF-κB p65 were dramatically decreased following ALA treatment (Fig. 6K, L). Significantly higher occludin levels in the ALA group also implied a milder inflammatory response in the intestine (Fig. 6K, M). These results suggested that ALA was able to protect hosts from the intense intestinal inflammation caused by *T. gondii* cyst infection by inhibiting the MyD88/NF-κB pathway.

### FMT from ALA-treated donor mice reshaped the gut microbiota and mitigated the colon inflammatory response caused by *T. gondii* infection

**Fig. 7:**
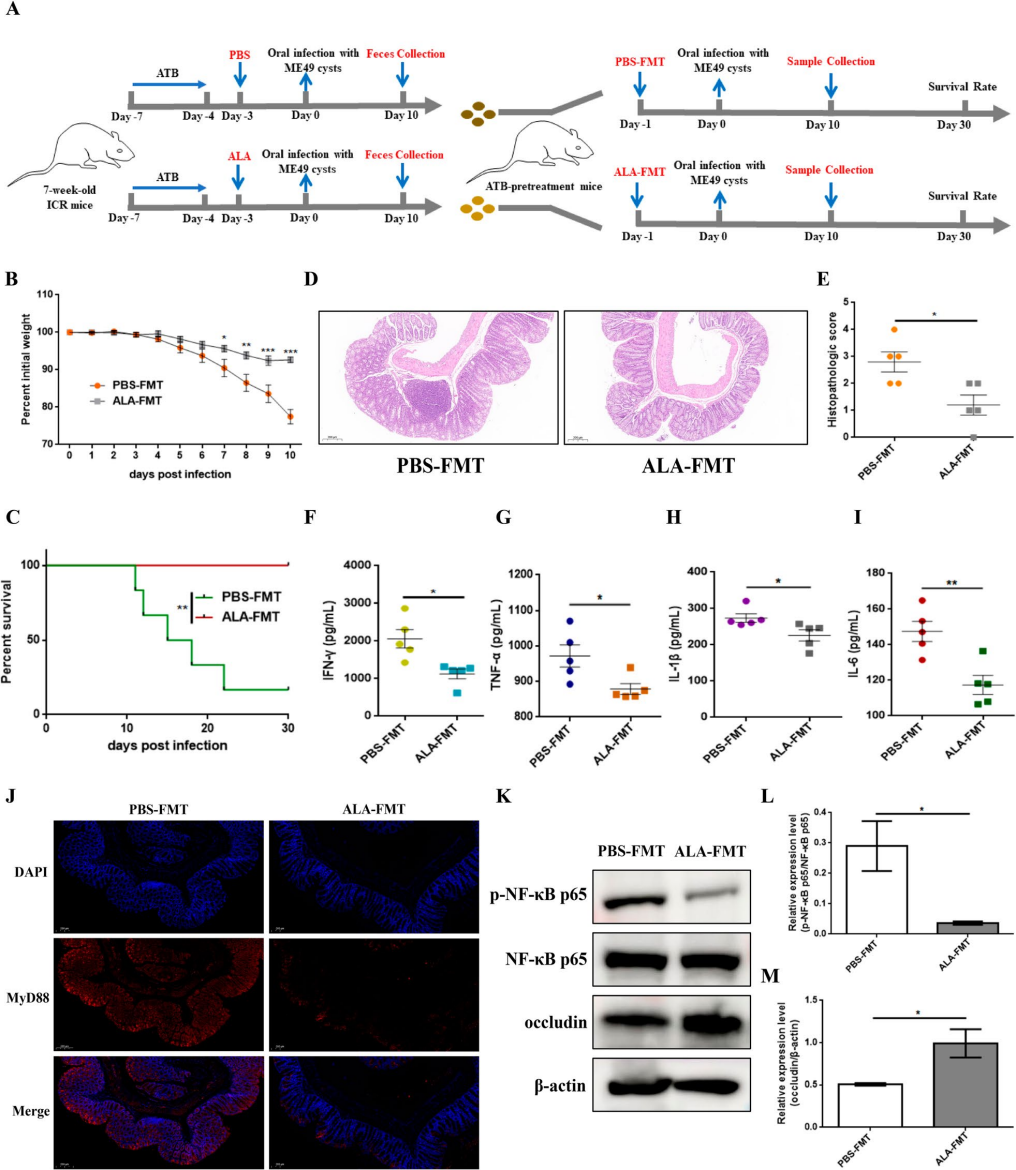
ALA-FMT mitigated colitis induced by *T. gondii* cyst infection. **A** Diagram of FMT experiments. **B** Mouse weight loss. **C** Mouse survival rate (*n* = 6 per group). Statistical significance was determined using two-way ANOVA. ***p < 0.001, **p < 0.01, *p < 0.05 compared with the PBS-FMT group. The survival rate was determined using the log-rank (Mantel-Cox) test. **p < 0.01 compared with the PBS-FMT group. **D**–**E** H&E staining of the mouse (*n* = 5 per group) colon tissues (scale bars = 200 μm, × 10 objective). Representative images are shown. **F**–**I** The levels of IFN-γ, TNF-α, IL-1β, and IL-6 were measured by ELISA (*n* = 5 per group). Statistical significance was determined with Student’s* t* test. **p < 0.01, *p < 0.05. **J** The expression level of MyD88 was measured by immunofluorescence. MyD88 protein was labelled with a red fluorophore, and the cell nucleus was labelled with a blue fluorophore (Scale bars = 200 μm, × 10 objective). Representative images are shown. **K**–**M** The expression levels of p-NF-κB p65 and occludin were measured by Western blotting. β-actin was used as a control. The data are presented as the means ± SEM. Statistical significance was determined with Student’s* t* test. *p < 0.05. All experimental data are representative of three independent experiments with similar results

**Fig. 8:**
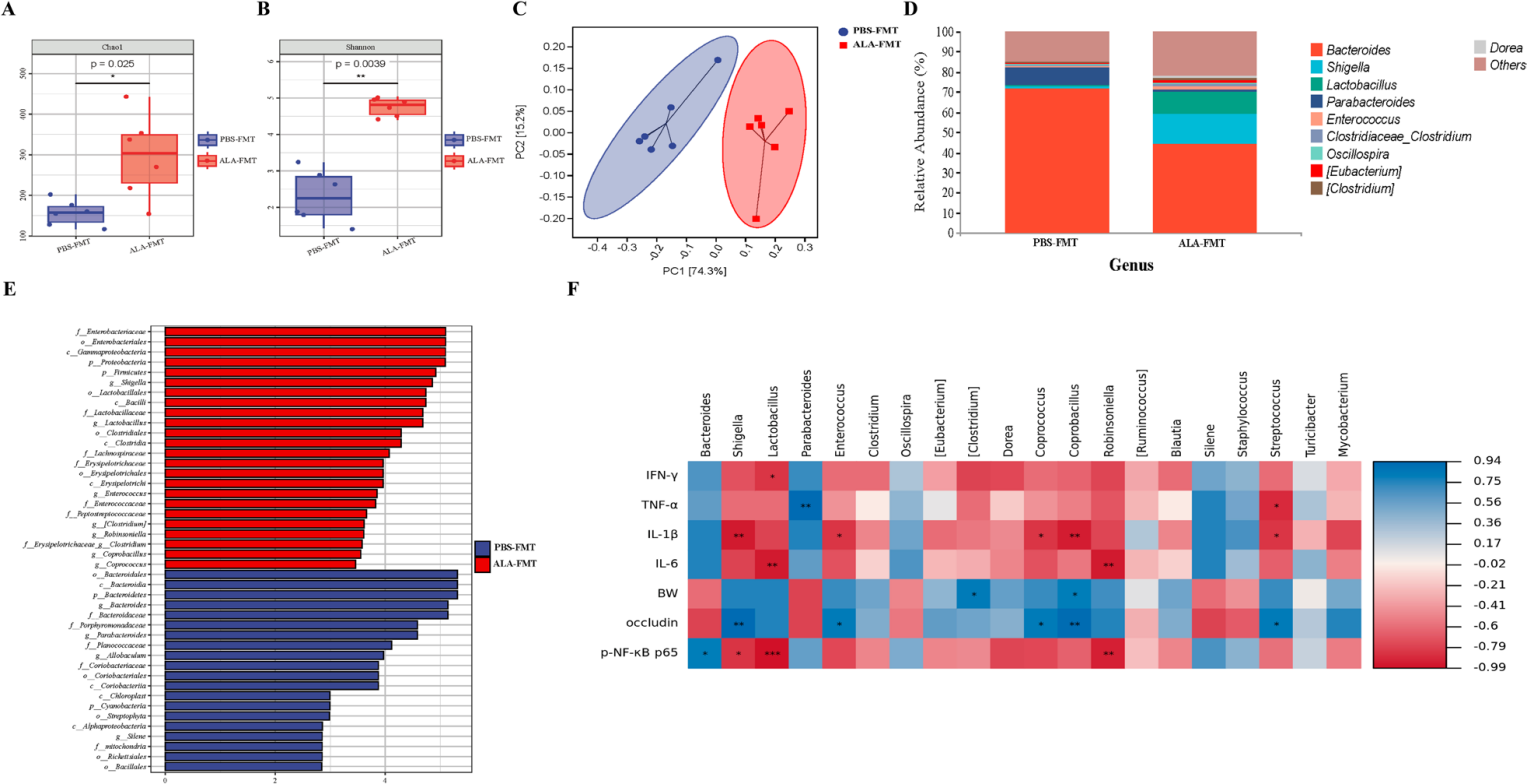
Composition of the fecal microbiota in FMT-treated mice. **A** Microbial community abundance was measured by the Chao1 index. **B** Microbial community diversity was measured by the Shannon index. **C** Principal coordinate analysis (PCoA) based on the weighted UniFrac distance matrix. **D** Relative abundances of fecal bacteria at the genus level. **E** Differentially abundant taxa of fecal microbiota between mice that underwent PBS-FMT and ALA-FMT were analyzed by LEfSe. LDA score ≥ 2. **F** Heatmap of Spearman’s correlation between gut microbiota abundance and phenotype/inflammatory factors. The colors range from blue (negative correlation) to red (positive correlation). ***p < 0.001, **p < 0.01, *p < 0.05

The gut microbiota coevolved with the intestinal immune system of the hosts, which had an important influence on the expression of immune mediators in the intestinal immune response [17]. To determine whether ALA inhibited intestinal inflammation caused by *T. gondii* infection by affecting the gut microbiota, fresh feces from the PBS and ALA groups were harvested and transplanted into ATB-pretreatment mice (Fig. 7A). The body weight was monitored daily, and the results in Fig. 7B, C revealed that FMT from ALA-treated donor mice significantly improved the body weight and increased the survival rate of recipient mice. Additionally, histopathological examination and ELISA were performed to evaluate intestinal inflammation, and the results showed that treatment with feces from the ALA group alleviated *T. gondii* cyst infection-induced intestinal injury compared with that in the PBS-FMT group (Fig. 7D–I). To investigate the molecular mechanism by which FMT from ALA-treated donor mice alters gut microbiota and relieves intestinal inflammation, immunofluorescence and Western blot analysis were used to detect the activation of the MyD88/NF-κB pathway. The protein levels of MyD88 in colon tissues in ALA-FMT group were significantly decreased compared with those in the PBS-FMT group (Fig. 7J). Meanwhile, the expression levels of p-NF-κB p65 showed a significant decrease in the ALA-FMT group (Fig. 7K, L), and occludin expression was dramatically upregulated (Fig. 7K, M). To further probe whether gut microbiota-related metabolite resist *Toxoplasma* infection by maintaining microbiota homeostasis, we next examined the fecal microbiota compositions in the FMT experiment using 16S rRNA gene sequencing. The Chao1 and Shannon indices of α-diversity manifested that the α-diversity of ALA-FMT-treated mice markedly increased, compared with that of PBS-FMT-treated mice (Fig. 8A, B). The PCA revealed that there was a significant difference in gut microbiota structure between the PBS-FMT and ALA-FMT groups (Fig. 8C). Microbiota compositions were considerably different between groups. At the phylum level, *Bacteroidetes*, *Proteobacteria*, and *Firmicutes* were the primary phyla in both groups. In addition, the relative abundance of *Proteobacteria* and *Firmicutes* increased in the ALA-FMT group, while that of *Bacteroidetes* was reduced compared with that in the PBS-FMT group (Fig. S6A). At the family level, *Enterobacteriaceae* and *Lactobacillaceae* were more enriched in the ALA-FMT mice (Fig. S6B). FMT from the ALA group observably upregulated the relative abundance of *Shigella*, *Lactobacillus*, and *Enterococcus* at the genus level (Fig. 8D, Fig. S7). Next, LEfSe analysis was performed to further uncover that all taxonomic levels of microbiota differed in the PBS-FMT and ALA-FMT groups, and the results showed that *Enterobacteriaceae*, *Proteobacteria*, *Shigella*, *Lactobacillus*, and *Enterococcus* were abundant in the mice treated with ALA feces, and *Bacteroides*, *Parabacteroides*, and *Allobaculum* were enriched in the PBS-FMT group (Fig. 8E). Overall, the above data suggested that FMT from ALA-treated donor mice could mitigate colitis induced by *T. gondii* infection, which is likely mediated, in part, by improving gut microbiota structure. In addition, Spearman correlation analysis was utilized to analyze the correlation between inflammation-related factors, body weight, and gut microbiota. As shown in Fig. 8F, the abundance of *[Clostridium]* and *Coprobacillus* was significantly (*P* < 0.05) and positively correlated with body weight and occludin, whereas the abundance of *Shigella*, *Lactobacillus*, *Robinsoniella*, and *Streptococcus* (*P* < 0.05) was significantly and negatively correlated with these inflammatory factors.

## Discussion

Oral infection is the primary transmission route of *T. gondii*. *T. gondii* enters the intestine and changes the composition of the gut microbiota, leading to acute and lethal intestinal inflammation. However, there is a lack of safe and effective drugs to treat intestinal inflammation caused by *T. gondii* infection. Growing evidence reveals the role of gut microbiota in the development and progression of toxoplasmosis. Significant changes occur in the composition and number of gut microbiota during parasite infection, and the modulation of the host immune response by immunomodulatory elements of the intestinal microorganism alters the resistance to *T. gondii* [13]. However, how the gut microbiota regulates intestinal inflammation, induced by *T. gondii* infection, remains poorly understood. Hence, we employed a mouse model of fecal microbiota transplantation to determine the potential gut microbiota and metabolites and expound the possible mechanisms underlying the resistance of candidates to oral infection of cysts. We identified a metabolite, ALA, that effectively alleviated *T. gondii*-induced lethal colitis and showed that its therapeutic effect was mediated by inhibiting the secretion of pro-inflammation cytokines through the inhibition of the MyD88/NF-κB pathway.

In the present study, mice infected with Δ*α-amy* cysts exhibited a disparate toxoplasmosis course and different intestinal pathological changes compared with that in WT cysts, and we reported for the first time that these differences were closely relevant to gut microbiota. IFN-γ produced by CD4 + Th1 cells is regulated by the MyD88 signalling pathway inherent to T cells in the intestinal mucosal immune response to *T. gondii* infection [44]. Although IFN-γ restrains parasite growth, it can trigger intense Th1-type cell immunity that exacerbates intestinal pathological damage and homeostasis dysregulation, thereby affecting host survival [14, 45]. Infection with ME49 cysts has been reported to cause massive necrosis of intestinal villi and mucosal cells in mice, leading to inflammatory bowel disease [9]. This is consistent with our present findings, and we also noticed that Δ*α-amy* cyst infection exhibited lower intestinal inflammation than ME49 cyst infection. There is a complex interaction between the mucosal immune system and gut microbiota during *T. gondii* infection, and changes in intestinal microorganisms occur in both acute and chronic infection stages [46]. The gut microbiota plays a dual role in activating host immune cells against *T. gondii* infection and inducing an inflammatory response in the intestine [47]. We hypothesize that the mitigation of intestinal inflammation may be due to a decrease in opportunistic pathogens caused by Δ*α-amy* cysts infection that controls the Th1-type immune response, thereby inhibiting the transmission of parasites and prolonging host survival.

*T. gondii* infection can trigger intestinal dysbiosis, especially the simultaneous loss of *Bacteroidetes* and the expansion of *Shigella* [14], which apparently corresponds with our results. In addition, the expansion of *Bacteroidetes* and remarkable reduction of *Enterobacteriaceae* in mice infected with Δ*α-amy* cysts, imply that *Bacteroidetes* may be probiotics that protect the host from *Toxoplasma* infection. Disparate species of gut microbiota may be beneficial or detrimental to hosts infected with parasites, and therefore gut commensal bacteria can be used to treat parasitosis. The ingestion of probiotics and FMT result in changes in the composition of gut microbiota, thereby altering the metagenome, transcriptome, proteome, and metabolome structure of gut microbiota [48, 49]. An increasing number of studies have shown that probiotics regulate the mucosal immune system by binding to Toll-like receptors (TLRs) and restraining the NF-κB pathway, which is responsible for the upregulation of the anti-inflammatory cytokine IL-10 and the downregulation of the pro-inflammatory cytokine TNF-α [50]. Increased bacterial diversity may have a therapeutic effect on chronic inflammatory bowel diseases and other autoimmune diseases in hosts coinfected with multiple parasitic protozoa [51]. Furthermore, the use of probiotics increases the diversity of gut microbiota and restores their balance, facilitating the recovery of inflammatory bowel diseases. Notably, a probiotic-rich dairy product markedly increases the relative abundance of *Lachnospiraceae* and *Akkermansia muciniphila* in the mouse intestine to control *T. gondii* infection [20]. Intriguingly, some potential probiotics were identified in our study for possible application in the treatment of toxoplasmosis, including *Pediococcus*, *Paraprevotella*, and *Macellibacteroides*, which were also highly positively correlated with the subsequently identified metabolites.

The interaction between parasites and gut microbiota may be regulated by the production of metabolites derived from gut microbiota that affect the survival and physiological state of parasites, leading to different infection processes [18, 52]. Importantly, some recent studies have confirmed that oral infection with *T. gondii* contributes to alterations in tryptophan metabolism, thereby reducing hyaluronic acid formation and exacerbating the intestinal inflammatory response [53]. Based on conjoint analysis, we identified that ALA associated with gut bacteria, a kind of ω-3 polyunsaturated fatty acid, has been proven to contribute to mitigating intestinal inflammation, which is mediated by promoting the differentiation of M2-type macrophages [28]. More importantly, recent studies also revealed that ALA can influence the inflammatory process by regulating gut microbiota [54–56]. Nevertheless, there is no report on the regulatory effect of ALA on inflammation caused by *T. gondii* infection. Our present results suggest that gut microbiota-related metabolite ALA can attenuate cyst infection-induced intestinal inflammation by regulating the colonization of specific gut microbial communities.

TLRs are one of the pattern recognition receptors of the innate immune system, and an imbalance in regulation can exacerbate mucosal injury in acute inflammation [57]. Intestinal innate immune responses triggered by external stimuli can upregulate the expression of TLRs, and deliver information about inflammatory responses through MyD88-dependent pathways, mediating the expression and release of inflammatory factors and inducing inflammatory injury [58]. It has also shown that mucosal responses to *T. gondii* infection are modulated by the TLR11-activated MyD88 signalling pathway in murine toxoplasmosis [35]. IFN-γ-dependent Paneth cell death is associated with uncontrolled expansion of gram-negative bacteria of the *Enterobacteriaceae* family [14]. Additionally, *T. gondii* infection elicits MAPK phosphorylation, NF-κB nuclear translocation, and secretion of IL-8, which are indicated to be MyD88 dependent [59]. The inflammatory factors induced by NF-κB can further maintain and activate NF-κB and then exacerbate inflammatory injury. It has also been reported that ALA reduces oxidative stress and colitis by regulating NF-κB signalling [42, 60]. In our experiments, we found that treatment with ALA could significantly alleviate inflammatory intestinal injury by suppressing the MyD88/NF-κB pathway, further confirming the important role of ALA in the treatment of colitis induced by *T. gondii* infection. Furthermore, occludin was the first transmembrane tight junction protein discovered, and the decreased expression of occludin contributes to increased intestinal permeability and elevated lipopolysaccharide levels [61, 62]. More importantly, ALA treatment observably increased the expression levels of occludin, further implying the therapeutic effect of ALA on toxoplasmosis.

**Fig. 9:**
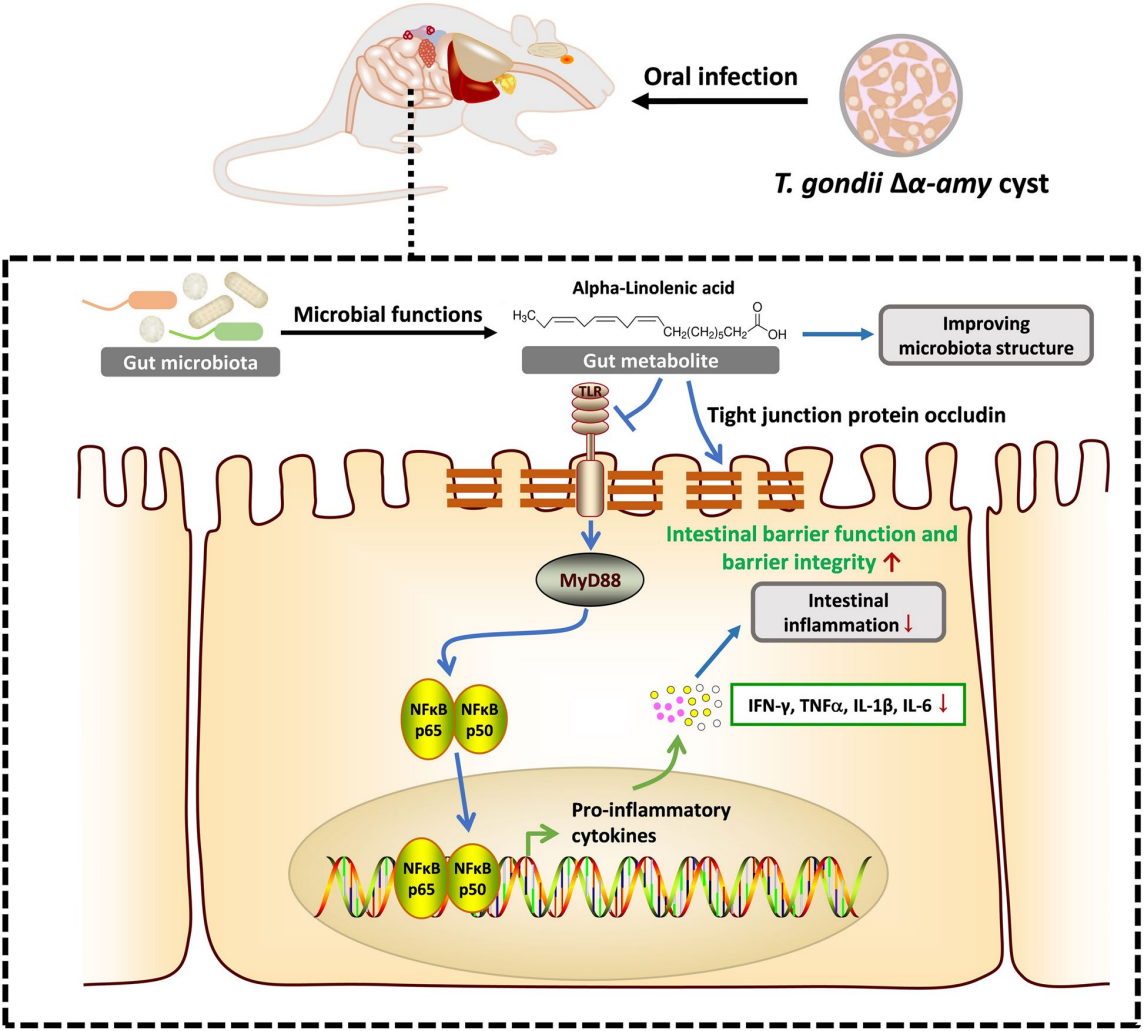
A schematic illustration of the proposed mechanism by which gut microbiota-related metabolite alpha-linolenic acid restrains intestinal inflammation induced by oral infection with *T. gondii*

In summary, we demonstrate that the gut microbiota is closely associated with the severity of *T. gondii* infection. Oral infection with avirulent Δ*α-amy* cysts influences the gut microbiota structure and is accompanied by changes in fatty acids such as ALA. Oral administration of ALA alleviated *T. gondii*-induced intestinal inflammation by improving the dysregulation of the gut microbiota and inhibiting the expression of inflammatory cytokines via the MyD88/NF-κB pathway (Fig. 9). Our present study elucidates the importance of gut microbiota and metabolites in the development of colitis induced by *T. gondii* infection and unveils the potential utility of ALA for the treatment of fatal intestinal inflammation caused by *Toxoplasma* infection.

### Supplementary Information

**Additional file 1: Fig. S1.** Brain cysts of ICR mice infected with the corresponding cysts. **P < 0.01, Student’s *t* test. The data are presented as the means ± SEM.

**Additional file 2: Fig. S2.** Principal component analysis (PCA) (A) and nonmetric multidimensional scaling (NMDS) score plot (B) between the ME49 and Δ*α-amy* groups.

**Additional file 3: Fig. S3.** Relative abundances of fecal bacterial between the ME49 and Δ*α-amy* groups at the phylum (A) and family (B) levels.

**Additional file 4: Fig. S4.** KEGG pathway analysis of differentially expressed genes between mice infected with ME49 and Δ*α-amy* cysts.

**Additional file 5: Fig. S5.** Metabolomic analysis of fecal samples from mice infected with ME49 and Δ*α-amy* cysts. A Total metabolome classifications of compounds with differential metabolites. B Fecal metabolome profiles were clustered using three-dimensional PLS-DA. Fecal metabolomic profiles were clustered using PCA with a boxplot (C) and three-dimensional PCA (D). The data are presented as the mean ± SEM. *P* values were determined using the nonparametric Kruskal-Wallis test.

**Additional file 6: Fig. S6.** Relative abundances of fecal bacteria between the PBS-FMT and ALA-FMT groups at the phylum (A) and family (B) levels.

**Additional file 7: Fig. S7.** Heatmap of the top 20 microbiota at the genus level in fecal samples from the PBS-FMT and ALA-FMT groups. Colour indicates the relative microbiota abundances in the group samples, and the corresponding relationship between the colour gradient and the value is shown in the gradient colour block.

## Data Availability

16S rRNA gene data has been presented in the National Center for Biotechnology Information (NCBI) Sequence Read Archive (SRA) database under accession number PRJNA951953 and PRJNA951528. Metabolomics raw data have been deposited in Mendeley Data (https://data.mendeley.com/datasets/kzgmy58n23/2).
