# Supplementary figures and images for: Gut microbiota-related metabolite alpha-linolenic acid mitigates intestinal inflammation induced by oral infection with Toxoplasma gondii
Source: Microbiome. 2023 Dec 12;11:273. doi: 10.1186/s40168-023-01681-0 (PMC10714487; doi:10.1186/s40168-023-01681-0)

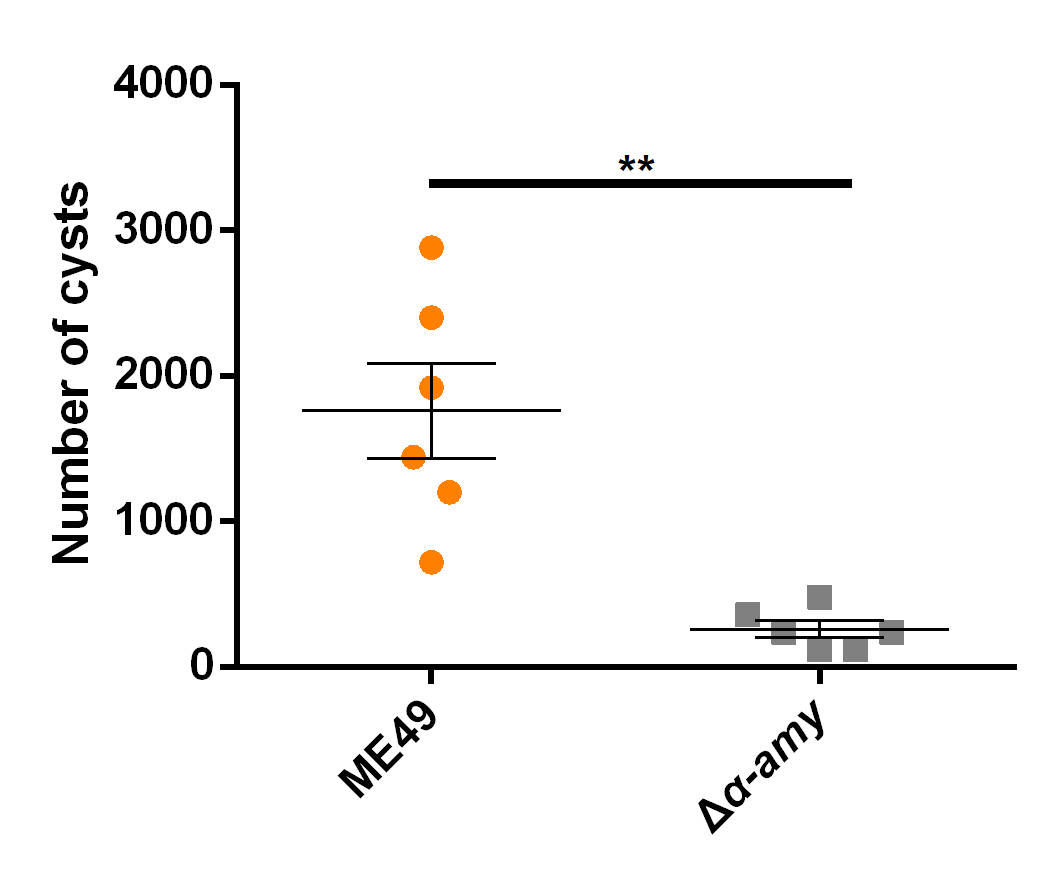

Supplement: Supplementary file 2 — Additional file 1: Fig. S1. Brain cysts of ICR mice infected with the corresponding cysts. **P < 0.01, Student’s t test. The data are presented as the means ± SEM. [file 40168_2023_1681_MOESM1_ESM.jpg]

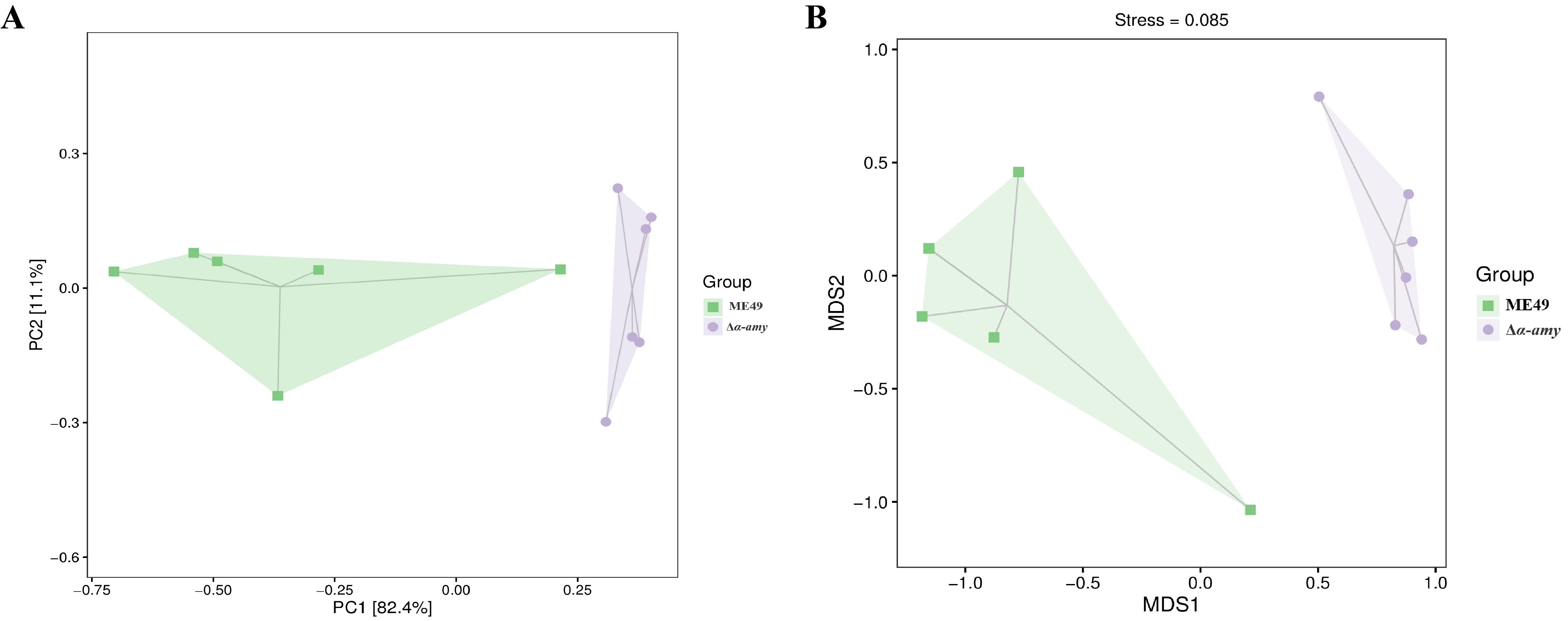

Supplement: Supplementary file 3 — Additional file 2: Fig. S2. Principal component analysis (PCA) (A) and nonmetric multidimensional scaling (NMDS) score plot (B) between the ME49 and Δα-amy groups. [file 40168_2023_1681_MOESM2_ESM.jpg]

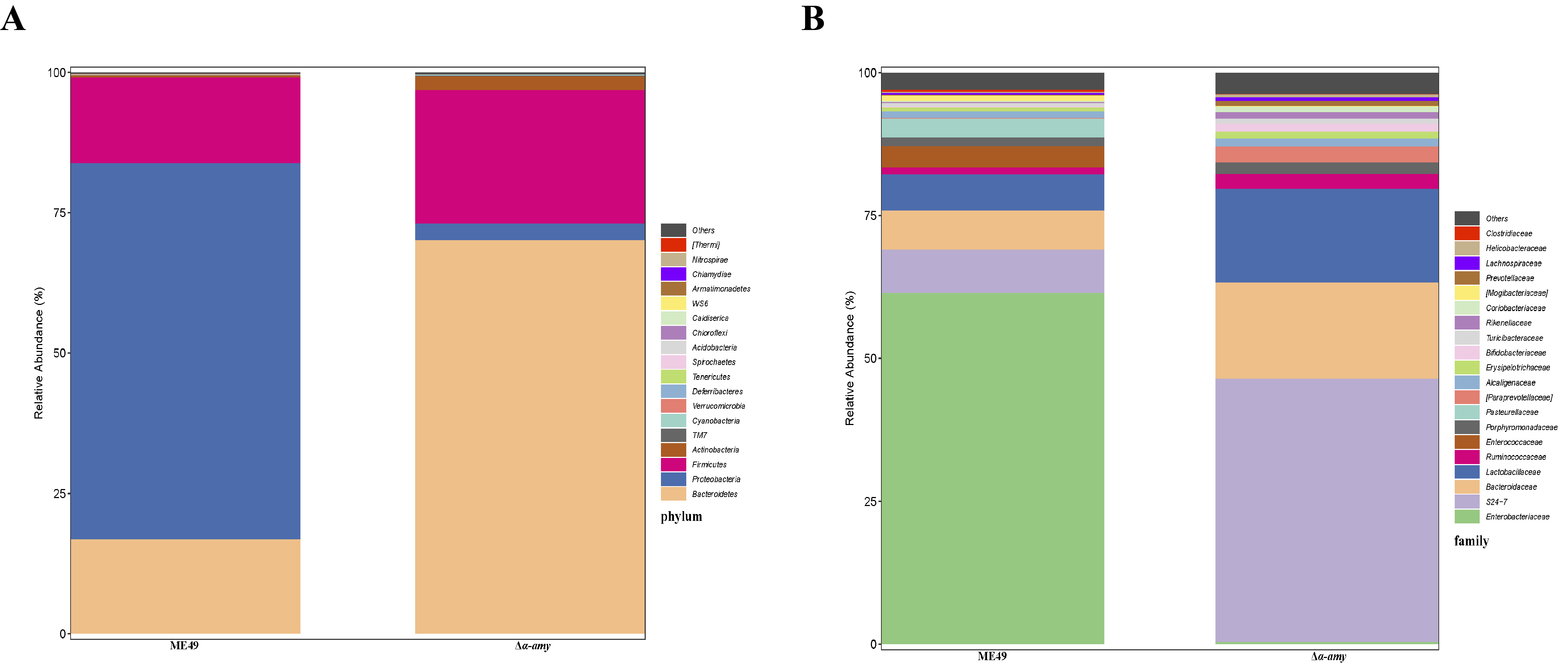

Supplement: Supplementary file 4 — Additional file 3: Fig. S3. Relative abundances of fecal bacterial between the ME49 and Δα-amy groups at the phylum (A) and family (B) levels. [file 40168_2023_1681_MOESM3_ESM.jpg]

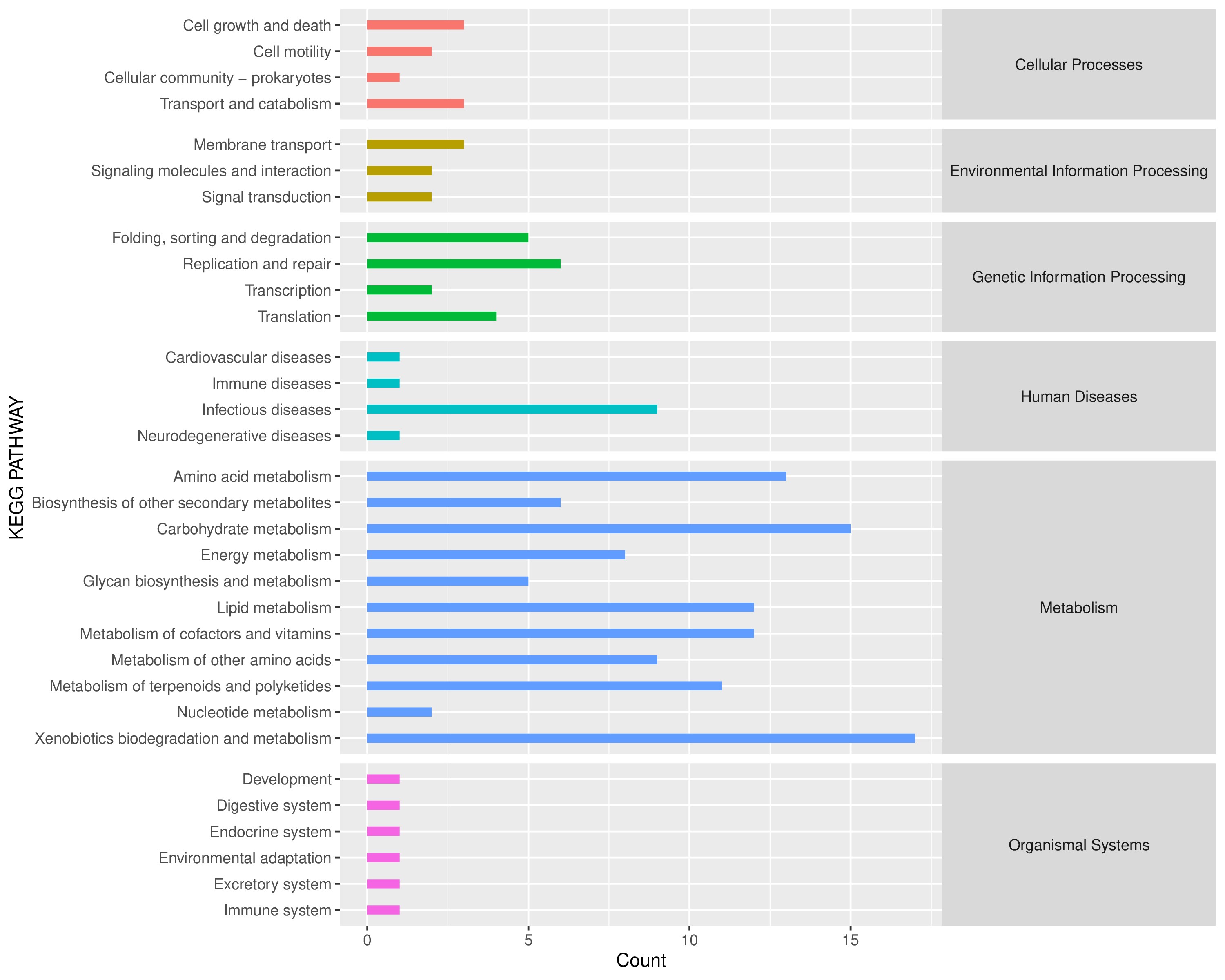

Supplement: Supplementary file 5 — Additional file 4: Fig. S4. KEGG pathway analysis of differentially expressed genes between mice infected with ME49 and Δα-amy cysts. [file 40168_2023_1681_MOESM4_ESM.jpg]

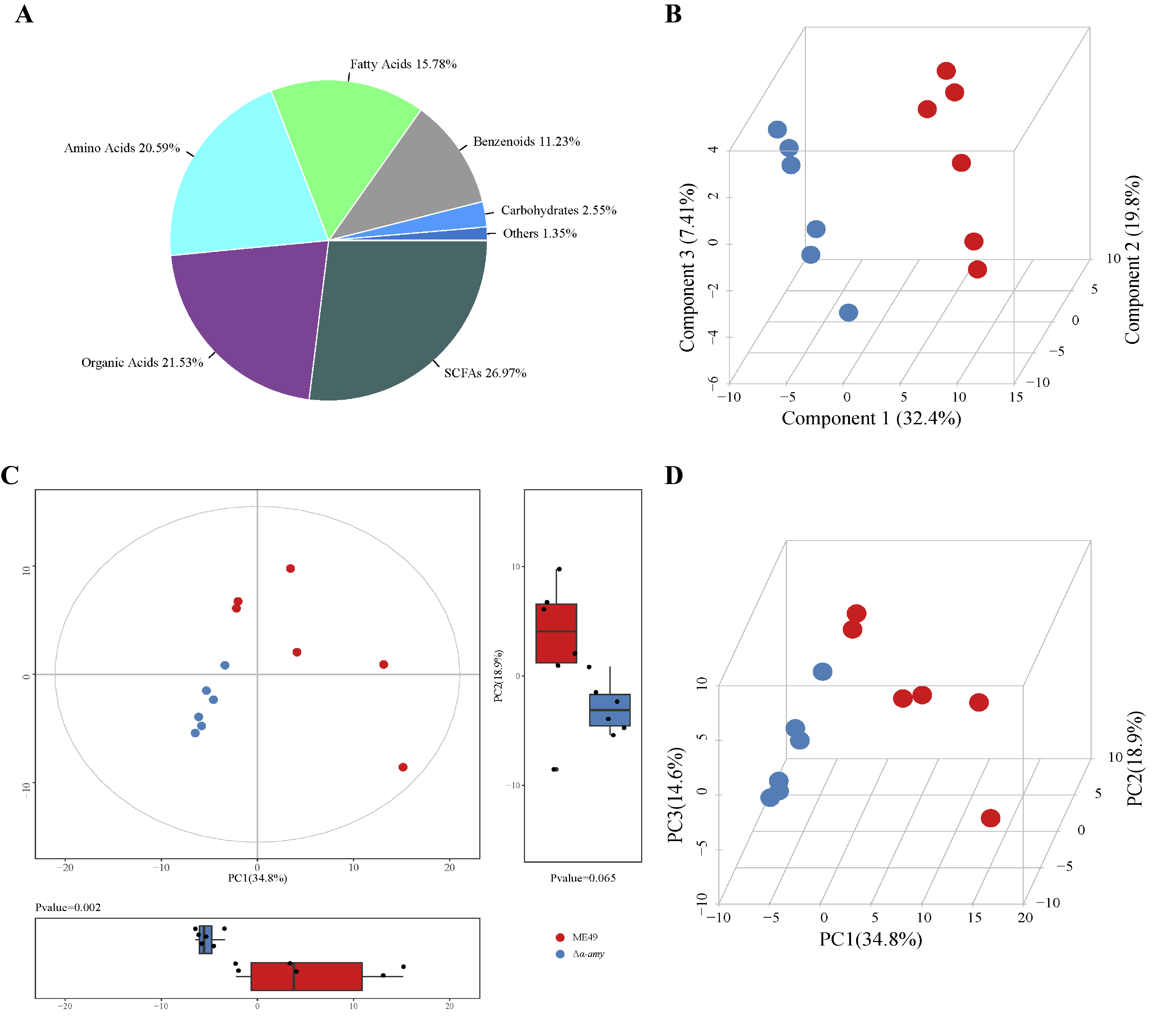

Supplement: Supplementary file 6 — Additional file 5: Fig. S5. Metabolomic analysis of fecal samples from mice infected with ME49 and Δα-amy cysts. A Total metabolome classifications of compounds with differential metabolites. B Fecal metabolome profiles were clustered using three-dimensional PLS-DA. Fecal metabolomic profiles were clustered using PCA with a boxplot (C) and three-dimensional PCA (D). The data are presented as the mean ± SEM. P values were determined using the nonparametric Kruskal-Wallis test. [file 40168_2023_1681_MOESM5_ESM.jpg]

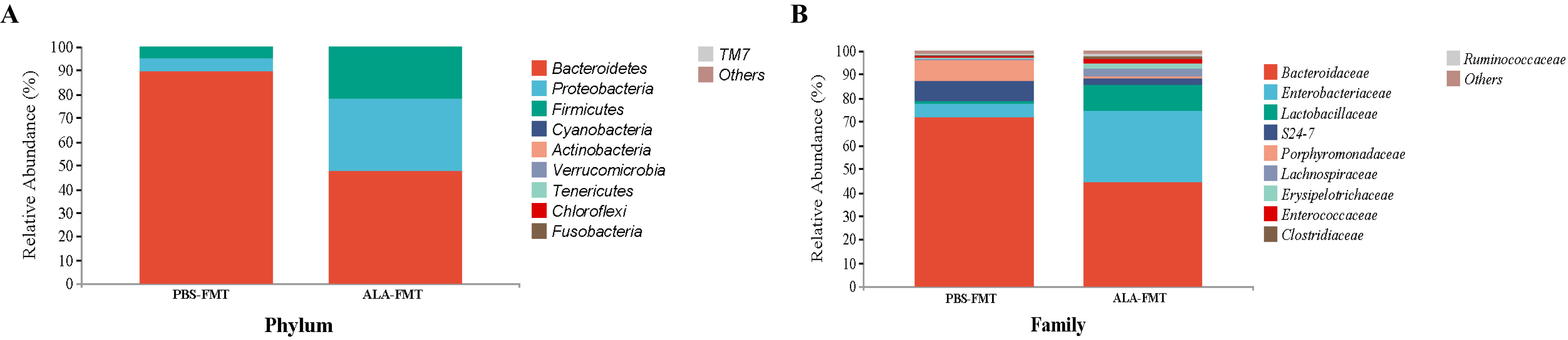

Supplement: Supplementary file 7 — Additional file 6: Fig. S6. Relative abundances of fecal bacteria between the PBS-FMT and ALA-FMT groups at the phylum (A) and family (B) levels. [file 40168_2023_1681_MOESM6_ESM.jpg]

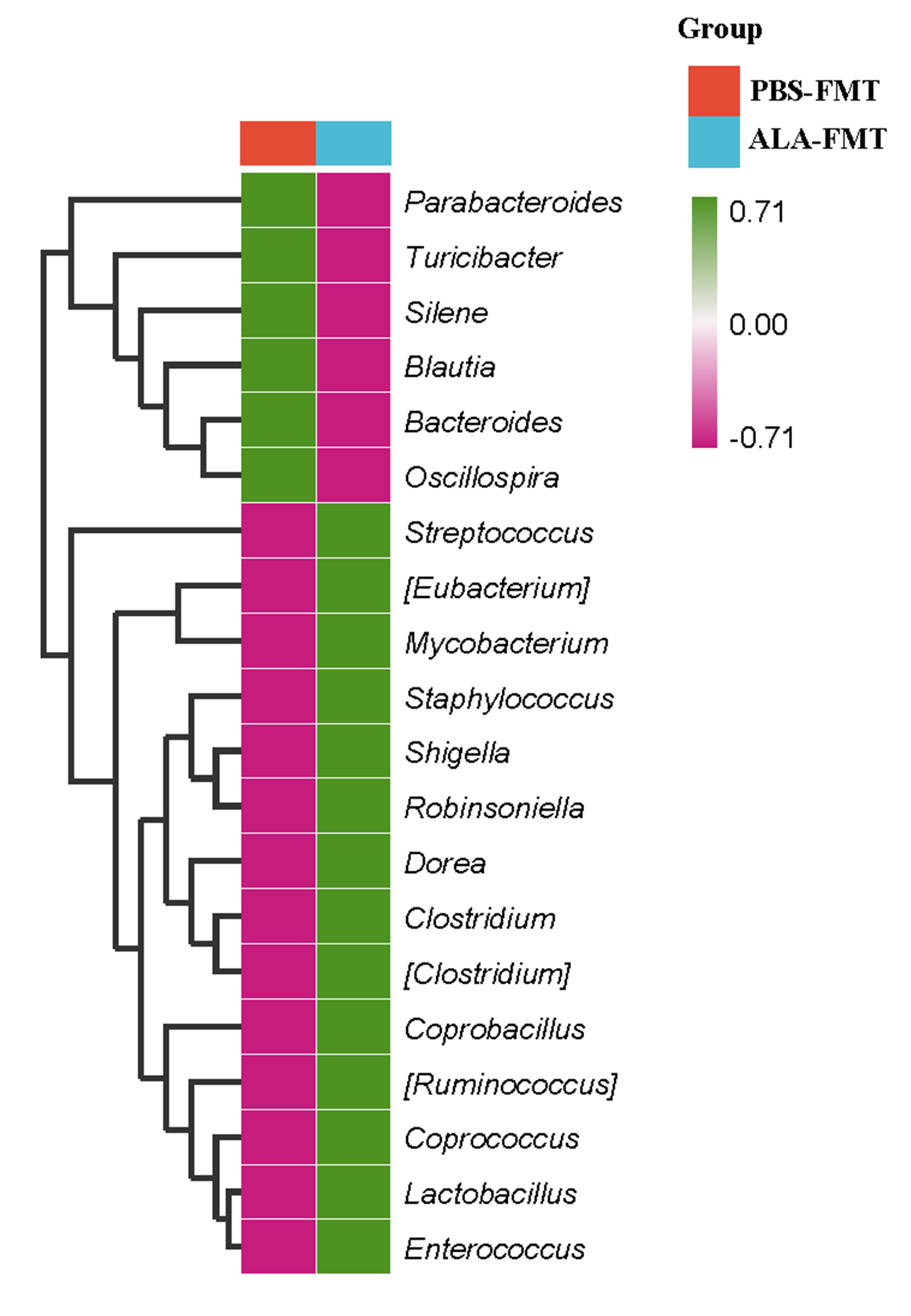

Supplement: Supplementary file 8 — Additional file 7: Fig. S7. Heatmap of the top 20 microbiota at the genus level in fecal samples from the PBS-FMT and ALA-FMT groups. Colour indicates the relative microbiota abundances in the group samples, and the corresponding relationship between the colour gradient and the value is shown in the gradient colour block. [file 40168_2023_1681_MOESM7_ESM.jpg]
